# Tailoring Fibroblast-Activation Protein Targeting for Theranostics: A Comparative Preclinical Evaluation of the ^68^Ga- and ^177^Lu-Labeled Monomeric and Dimeric Fibroblast-Activation Protein Inhibitors DOTA.SA.FAPi and DOTAGA.(SA.FAPi)_2_

**DOI:** 10.3390/molecules29133093

**Published:** 2024-06-28

**Authors:** Tilman Läppchen, Adrianna Bilinska, Eirinaios Pilatis, Elena Menéndez, Surachet Imlimthan, Euy Sung Moon, Ali Afshar-Oromieh, Frank Rösch, Axel Rominger, Eleni Gourni

**Affiliations:** 1 Department of Nuclear Medicine, Inselspital, Bern University Hospital, 3010 Bern, Switzerland; tilman.laeppchen@insel.ch (T.L.); adrianna.bilinska@extern.insel.ch (A.B.); eirinaios.pilatis@insel.ch (E.P.); elena.menendez@insel.ch (E.M.); surachet.imlimthan@helsinki.fi (S.I.); ali.afshar@insel.ch (A.A.-O.); axel.rominger@insel.ch (A.R.); 2 Department of Chemistry—TRIGA Site, Johannes Gutenberg-University Mainz, 55128 Mainz, Germany; esmoon92@googlemail.com (E.S.M.); froesch@uni-mainz.de (F.R.)

**Keywords:** fibroblast activation protein inhibitors (FAPi), FAPi-monomer, FAPi-dimer, gallium-68, lutetium-177

## Abstract

Background: FAP radiopharmaceuticals show promise for cancer diagnosis; however, their limited tumor residency hinders treatment. This study compared two FAPi derivatives, DOTA.SA.FAPi and DOTAGA.(SA.FAPi)_2_, labeled with gallium-68 and lutetium-177, aiming to determine an optimum combination for creating theranostic pairs. Methods: The radiotracers were studied for lipophilicity, binding to human serum proteins, and binding to human cancer-associated fibroblasts (CAFs) in vitro, including saturation and internalization/externalization studies. PET/SPECT/CT and biodistribution studies were conducted in PC3 and U87MG xenografts for [^68^Ga]Ga-DOTA.SA.FAPi and [^68^Ga]Ga-DOTAGA.(SA.FAPi)_2_. [^177^Lu]Lu-DOTA.SA.FAPi and [^177^Lu]Lu-DOTAGA.(SA.FAPi)_2_, were evaluated in PC3 xenografts. Biodistribution studies of [^68^Ga]Ga-DOTA.SA.FAPi were performed in healthy male and female mice. Results: All radiotracers exhibited strong binding to FAP. Their internalization rate was fast while only [^177^Lu]Lu-DOTAGA.(SA.FAPi)_2_ was retained longer in CAFs. [^68^Ga]Ga-DOTAGA.(SA.FAPi)_2_ and [^177^Lu]Lu-DOTAGA.(SA.FAPi)_2_ displayed elevated lipophilicity and affinity for human serum proteins compared to [^68^Ga]Ga-DOTA.SA.FAPi and [^177^Lu]Lu-DOTA.SA.FAPi. In vivo studies revealed slower washout of [^68^Ga]Ga-DOTAGA.(SA.FAPi)_2_ within 3 h compared to [^68^Ga]Ga-DOTA.SA.FAPi. The tumor-to-tissue ratios of [^68^Ga]Ga-DOTAGA.(SA.FAPi)_2_ versus [^68^Ga]Ga-DOTA.SA.FAPi did not exhibit any significant differences. [^177^Lu]Lu-DOTAGA.(SA.FAPi)_2_ maintained a significant tumor uptake even after 96 h p.i. compared to [^177^Lu]Lu-DOTA.SA.FAPi. Conclusions: Dimeric compounds hold promise for therapy, while monomers are better suited for diagnostics. Finding the right combination is essential for effective disease management.

## 1. Introduction

One emerging and highly promising class of theranostic radiopharmaceuticals is based on fibroblast-activation protein (FAP) targeting. FAP is a membrane-bound serine protease in the tumor microenvironment and has been shown to be implicated in various pathological conditions including cancer. It is abundantly expressed in the stroma of many solid tumors, including pancreatic, lung, colon, prostate cancer, and others [1,2]. Numerous classes of tailored and potent agents for cancer diagnosis, therapy monitoring, and treatment have been extensively explored in preclinical settings [3,4,5,6,7,8,9,10,11,12,13,14,15,16,17,18]. Many of these have also undergone clinical investigation, demonstrating significant potential [3,7,8,9,17,18,19,20,21,22,23,24,25,26].

Various categories of theranostic FAP radiotracers can be designed by considering their structural characteristics and target specificity. When categorizing them, they can be classified as small-molecule FAP inhibitors (FAPi), peptides, and antibodies [1,2]. Although the diagnostic FAPi-based radiotracers offer numerous advantages, such as their clinically proven ability to detect a variety of cancer entities, their therapeutic counterparts present a set of challenges, making them a fascinating field of research. One of the main drawbacks of therapeutic FAPi-based radiotracers is their relatively short tumor residence time with biological half-lives much shorter than the physical half-lives of the therapeutic radionuclides lutetium-177 or actinium-225 [27]. This limits the exposure time of the tumor to radiation, compromising the effectiveness of the cancer treatment [3,8,20,28,29]. Several strategies have been pursued to increase the tumor residence time of therapeutic FAPi-based radiotracers, mainly focusing on improving their affinity and/or pharmacokinetics, by creating dimeric analogs or introducing albumin-binding moieties to the monomeric vectors [4,5,6,10,11,12,15,17].

A particular promising theranostic pair is the monomeric DOTA.SA.FAPi and the dimeric DOTAGA.(SA.FAPi)_2_, both based on the selective, high-affinity FAP inhibitor UAMC1110 ((S)-*N*-(2-(2-cyano-4,4-difluoropyrrolidin-1-yl)-2-oxoethyl)quinoline-4-carboxamide)), coupled to the respective chelator via a squaramide (SA) linker [12,13,14,30]. In contrast to the monomeric DOTA.SA.FAPi, which contains a single SA-linked UAMC110 unit coupled to the bifunctional chelator DOTA, the homodimer DOTAGA.(SA.FAPi)_2_, contains two identical SA-linked UAMC110 units coupled to the trifunctional chelator DOTAGA (Figure S1). Both compounds have already been labeled with gallium-68 and lutetium-177, and the resulting radiotracers have successfully been applied in the clinic [19,20,21,22,23,24,31]. Nevertheless, a thorough preclinical evaluation has not been performed so far.

In the current study, we set out to close this gap by conducting a comprehensive preclinical assessment of the ^68^Ga- and ^177^Lu-labeled DOTA.SA.FAPi and DOTAGA.(SA.FAPi)_2_. The resulting four radiotracers were characterized in terms of radiolabeling, in vitro stability, lipophilicity, saturation binding (Kd and Bmax), internalization and externalization in cells, ex vivo organ distribution and in vivo PET/SPECT/CT imaging in prostate and glioblastoma tumor-bearing mice.

## 2. Results

### 2.1. Radiolabeling/Quality Control of the Radiotracers/Stability

DOTA.SA.FAPi and DOTAGA.(SA.FAPi)_2_ (a schematical representation of both precursors can be found in the Supplementary Data in Figure S1) were successfully labelled with gallium-68 in >98% radiochemical purity. The apparent molar activity (A_m_) was 8.5 ± 0.8 GBq/µmol for [^68^Ga]Ga-DOTA.SA.FAPi and 12.4 ± 4.4 GBq/µmol for [^68^Ga]Ga-DOTAGA.(SA.FAPi)_2_. The non-decay corrected isolated radiochemical yield was 57 ± 9% for [^68^Ga]Ga-DOTA.SA.FAPi (*n* = 10) and 68 ± 8% for [^68^Ga]Ga-DOTAGA.(SA.FAPi)_2_ (*n* = 10). No formation of colloids was observed.

The labeling yields for the ^177^Lu-labeled conjugates were >95% with apparent molar activities of 7–20 GBq/μmol, depending on the study.

The formulated ^68^Ga-labeled and ^177^Lu-labeled radiotracers proved highly stable, as neither radiolysis nor chemical decomposition was observed for a period of 4 and 48 h post labeling, respectively.

### 2.2. Lipophilicity/Protein Binding Studies

The LogD_octanol/PBS-pH7.4_ data and the binding of the ^68^Ga- and ^177^Lu-labeled radiotracers to human serum proteins is listed in Table 1. With an about 2-fold higher LogD_octanol/PBS_ value, the dimeric [^68^Ga]Ga- and [^177^Lu]Lu-DOTAGA.(SA.FAPi)_2_ proved more lipophilic than their monomeric counterparts [^68^Ga]Ga- and [^177^Lu]Lu-DOTA.SA.FAPi.

Similarly, after 30 min of incubation with human serum, [^68^Ga]Ga- and [^177^Lu]Lu-DOTAGA.(SA.FAPi)_2_ showed about 2-fold higher protein binding compared to [^68^Ga]Ga- and [^177^Lu]Lu-DOTA.SA.FAPi.

### 2.3. Saturation Binding/Internalization/Externalization Studies

^68/nat^Ga-DOTA.SA.FAPi, ^68/nat^Ga-DOTAGA.(SA.FAPi)_2_, ^177/nat^Lu-DOTA.SA.FAPi and ^177/nat^Lu-DOTAGA.(SA.FAPi)_2_ exhibited similar affinities for CAFs, with K_d_ values of 0.82 ± 0.22 nM, 1.15 ± 0.26 nM, 1.60 ± 0.53 nM and 1.35 ± 0.69 nM, respectively (Figure S7). The B_max_ values were at the same level for all (0.41 ± 0.03 nM, 0.47 ± 0.03 nM, 0.74 ± 0.08 nM and 0.37 ± 0.05 nM, respectively), which corresponds to approximately 3 × 10^5^ receptors/cell.

**Figure 1:**
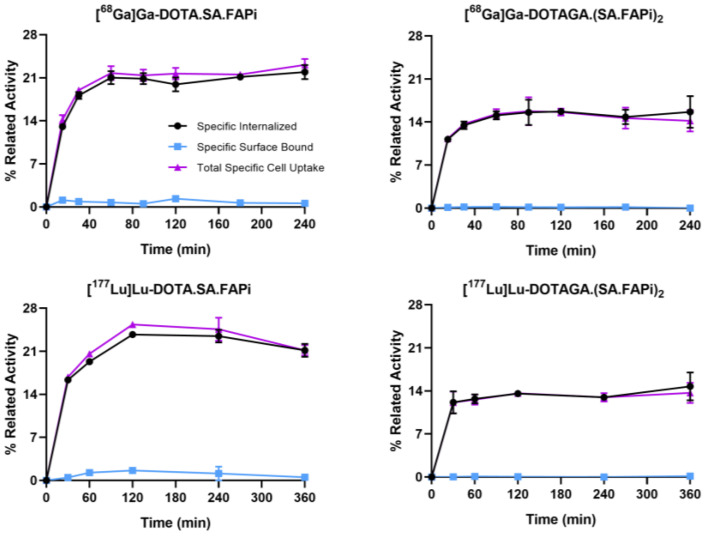
Internalization rate and specific surface bound uptake after the incubation of CAFs with [^68^Ga]Ga-DOTA.SA.FAPi, [^68^Ga]Ga-DOTAGA.(SA.FAPi)_2_, [^177^Lu]Lu-DOTA.SA.FAPi and [^177^Lu]Lu-DOTAGA.(SA.FAPi)_2_ within 4 and 6 h at 37 °C. Total specific cell uptake calculated as specific surface bound fraction plus specific internalized fraction. Total specific cell uptake is expressed as the percentage of the total applied radioactivity. Nonspecific binding was determined in the presence of 1 μM UAMC1110.

All of the radioligands were found to be well associated with CAFs within the incubation time (Figure 1). Continued exposure of CAFs to the radioligands resulted in a gradual increase of the total cell associated uptake. [^68^Ga]Ga-DOTA.SA.FAPi (21.9 ± 1.2%) and [^177^Lu]Lu-DOTA.SA.FAPi (21.1 ± 1.1%) exhibited higher values compared to [^68^Ga]Ga-DOTAGA.(SA.FAPi)_2_ (15.6 ± 2.6%) and [^177^Lu]Lu-DOTAGA.(SA.FAPi)_2_ (14.7 ± 2.3%) at 4 and 6 h, respectively (*p* = 0.0035 for [^68^Ga]Ga-DOTA.SA.FAPi and [^68^Ga]Ga-DOTAGA.(SA.FAPi)_2_ and *p* = 0.0031 for [^177^Lu]Lu-DOTA.SA.FAPi and [^177^Lu]Lu-DOTAGA.(SA.FAPi)_2_). All were internalized rapidly, with more than 95% of the total cell associated activity internalized in the cells at all tested time points.

**Figure 2:**
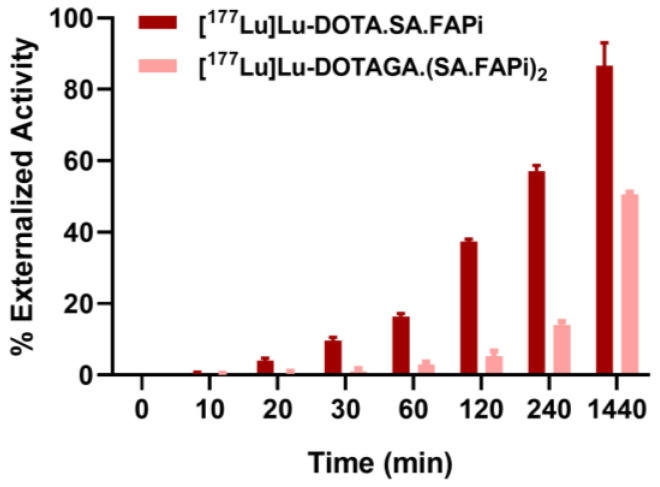
Percentage of the activity externalized from the cells expressed in relation to the total internalized activity (100%).

A side-by-side comparison of [^177^Lu]Lu-DOTA.SA.FAPi and [^177^Lu]Lu-DOTAGA.(SA.FAPi)_2_ regarding their externalization rate from CAFs showed that [^177^Lu]Lu-DOTAGA.(SA.FAPi)_2_ exhibited higher retention in CAFs compared to [^177^Lu]Lu-DOTA.SA.FAPi (Figure 2). After 24 h at 37 °C, 48% of the total internalized activity had remained in the CAFs for [^177^Lu]Lu-DOTAGA.(SA.FAPi)_2_, while at the same time point more than 93% of the total internalized activity had been released from the CAFs into the medium when [^177^Lu]Lu-DOTA.SA.FAPi was evaluated.

Blocking experiments performed with excess UAMC1110, showed negligible nonspecific binding on the CAFs’s surface, demonstrating high specificity of the radioligands toward FAP-positive CAFs (Figure S8).

### 2.4. Biodistribution Studies

#### 2.4.1. [^68^Ga]Ga-DOTA.SA.FAPi and [^68^Ga]Ga-DOTAGA.(SA.FAPi)_2_

**Figure 3:**
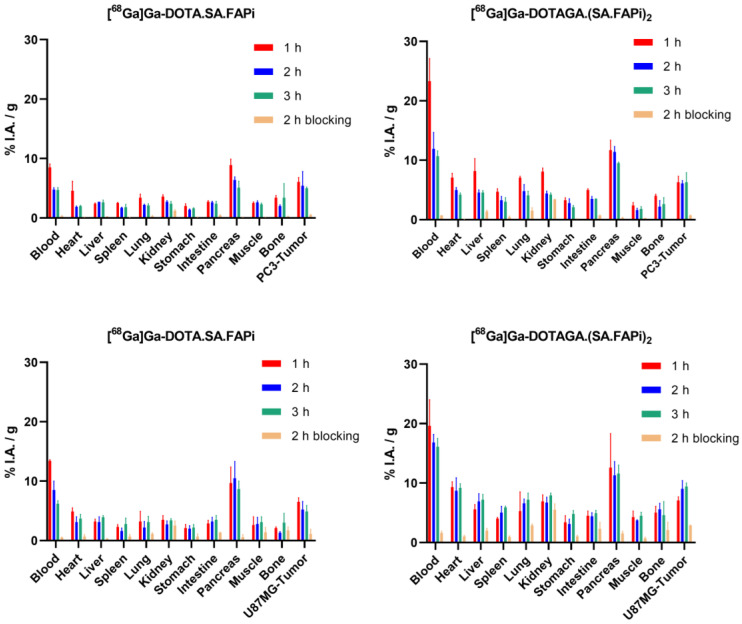
Biodistribution data of [^68^Ga]Ga-DOTA.SA.FAPi and [^68^Ga]Ga-DOTAGA.(SA.FAPi)_2_ in PC3 and U87MG xenografts at 1, 2, and 3 h p.i along with blocking studies data at 2 h p.i. Data have been calculated as %I.A./g of tissue and are presented as mean ± SD (*n* = 4).

**Figure 4:**
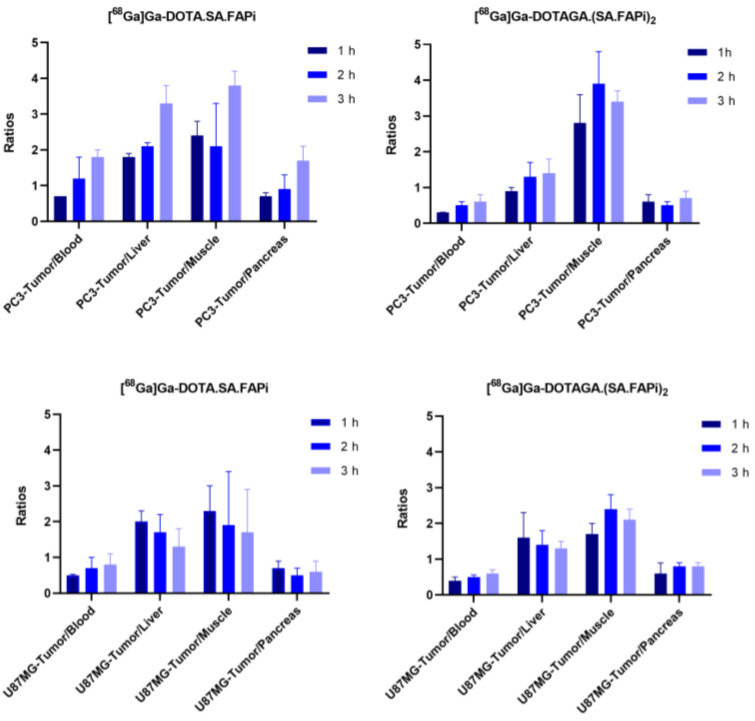
Tissue radioactivity ratios of [^68^Ga]Ga-DOTA.SA.FAPi and [^68^Ga]Ga-DOTAGA.(SA.FAPi)_2_ in PC3 and U87MG xenografts at 1, 2 and 3 h p.i.

**Figure 5:**
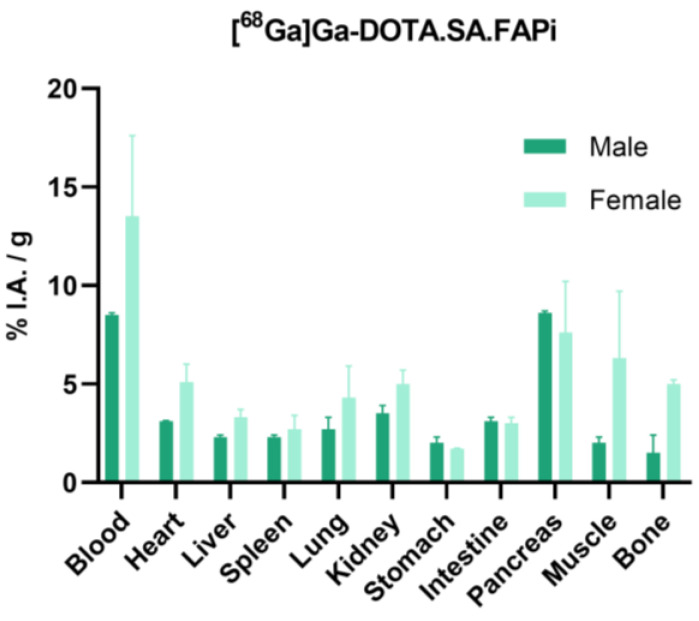
Biodistribution data of [^68^Ga]Ga-DOTA.SA.FAPi in healthy male and female mice at 1 h p.i. Data have been calculated as %I.A./g of tissue and are presented as mean ± SD (*n* = 4).

Ex vivo biodistribution data and tumor-to-tissue ratios of [^68^Ga]Ga-DOTA.SA.FAPi and [^68^Ga]Ga-DOTAGA.(SA.FAPi)_2_ in PC3 and U87MG tumor-bearing mice as well as ex vivo biodistribution data of [^68^Ga]Ga-DOTA.SA.FAPi in healthy male and female mice are depicted on Figure 3, Figure 4 and Figure 5, respectively. Detailed tables with the ex vivo biodistribution values and tumor-to-tissue ratios are also given in the Supplementary Data (Tables S1–S3). [^68^Ga]Ga-DOTA.SA.FAPi and [^68^Ga]Ga-DOTAGA.(SA.FAPi)_2_ exhibited high and persistent blood pool retention at all tested time points for both tumor-bearing mice; however, [^68^Ga]Ga-DOTAGA.(SA.FAPi)_2_ revealed significantly higher values than [^68^Ga]Ga-DOTA.SA.FAPi (*p* = 0.002, 0.0161, 0.004 at 1, 2 and 3 h p.i. for PC3 and *p* = 0.0051, 0.0018, 0.002 at 1, 2 and 3 h p.i. for U87MG). Blood clearance seemed to be somehow faster for PC3 compared to U87MG mice for both ^68^Ga-labeled radioligands. Likewise, the PC3 mice showed lower uptake in non-target organs including muscles, lung, salivary glands, and joints. For both tumor models, [^68^Ga]Ga-DOTA.SA.FAPi appears to be superior compared to [^68^Ga]Ga-DOTAGA.(SA.FAPi)_2_ in terms of tumor-to-background ratios in the course of 3 h.

Biodistribution studies of [^68^Ga]Ga-DOTA.SA.FAPi in healthy male and female mice at 1 h p.i. also indicated significantly higher background uptake in female compared to male mice (*p* = 0.0226, 0.0268, 0.0351 and 0.028 for muscle, lung, bone and blood).

#### 2.4.2. [^177^Lu]Lu-DOTA.SA.FAPi and [^177^Lu]Lu-DOTAGA.(SA.FAPi)_2_

**Figure 6:**
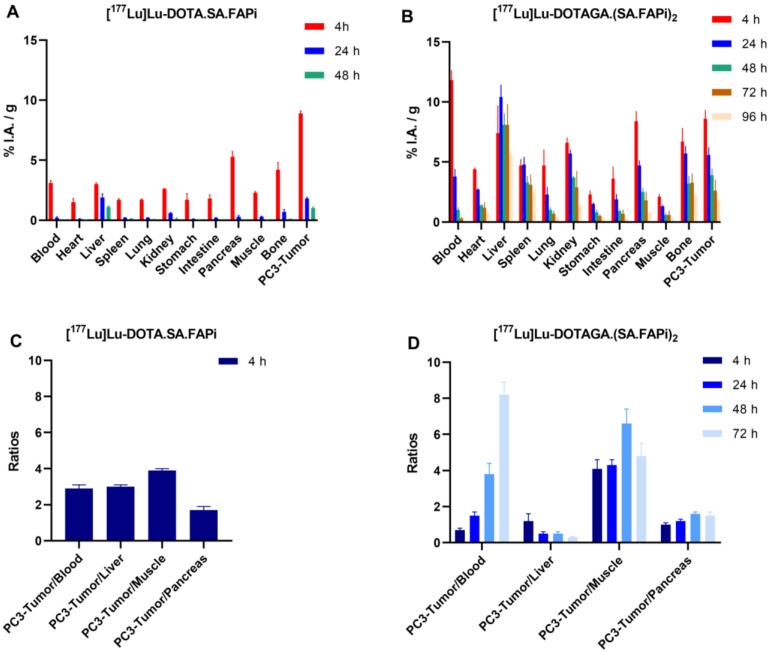
Biodistribution data of (**A**) [^177^Lu]Lu-DOTA.SA.FAPi at 4, 24 and 48 h p.i. and (**B**) [^177^Lu]Lu-DOTAGA.(SA.FAPi)_2_ at 4, 24, 48, 72 and 96 h p.i. on PC3 xenografts along with (**C**,**D**) the relevant tissue radioactivity ratios. Data have been calculated as %I.A./g of tissue and are presented as mean ± SD (*n* = 4).

Biodistribution data and tumor-to-tissue ratios of [^177^Lu]Lu-DOTA.SA.FAPi and [^177^Lu]Lu-DOTAGA.(SA.FAPi)_2_ in PC3 tumor-bearing mice are summarized in Figure 6A–D. Detailed tables with the ex vivo biodistribution values and tumor-to-tissue ratios are also given in the Supplementary Data (Table S4). Both are taken up by the PC3 tumors at early time points, exhibiting similar uptake (8.9 ± 0.2 and 8.6 ± 0.7%I.A./g for [^177^Lu]Lu-DOTA.SA.FAPi and [^177^Lu]Lu-DOTAGA.(SA.FAPi)_2_ at 4 p.i., respectively). The wash-out of the tumor-accumulated activity for [^177^Lu]Lu-DOTAGA.(SA.FAPi)_2_ was slower compared to [^177^Lu]Lu-DOTA.SA.FAPi. Even after 48 h p.i., the tumor uptake of [^177^Lu]Lu-DOTAGA.(SA.FAPi)_2_ was higher by a factor of four compared to [^177^Lu]Lu-DOTA.SA.FAPi. As was also observed in the case of the ^68^Ga-labeled radiotracers, the background uptake for [^177^Lu]Lu-DOTAGA.(SA.FAPi)_2_ was higher than for [^177^Lu]Lu-DOTA.SA.FAPi, resulting in lower tumor-to-background ratios at the early time points. Nevertheless, with 1.8 ± 0.1%I.A./g still being present in the tumor 96 h p.i., [^177^Lu]Lu-DOTAGA.(SA.FAPi)_2_ shows long and persistent tumor retention, ensuring a higher accumulated dose to the tumor compared to [^177^Lu]Lu-DOTA.SA.FAPi.

### 2.5. Small-Animal PET/SPECT/CT Studies

**Figure 7:**
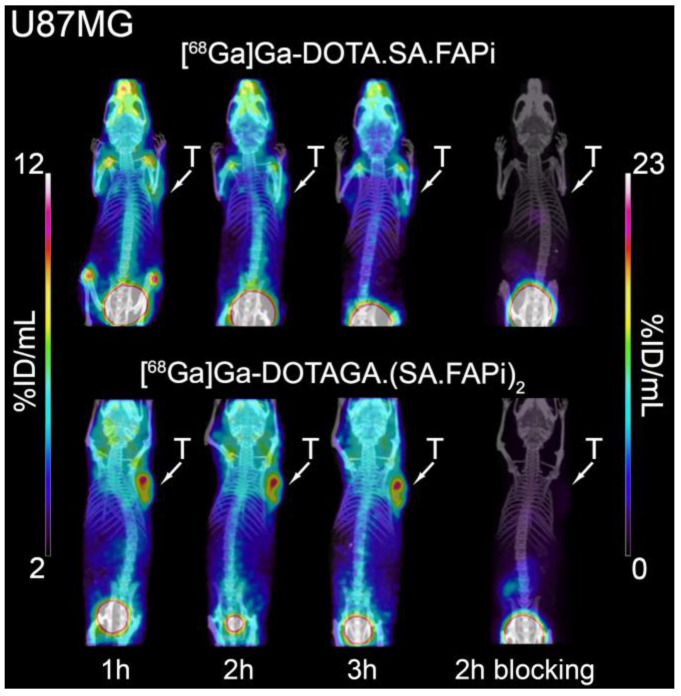
PET/CT images of U87MG tumor-bearing mice 1 h, 2 h, and 3 h after injection of [^68^Ga]Ga-DOTA.SA.FAPi and [^68^Ga]Ga-DOTAGA.(SA.FAPi)_2_.

**Figure 8:**
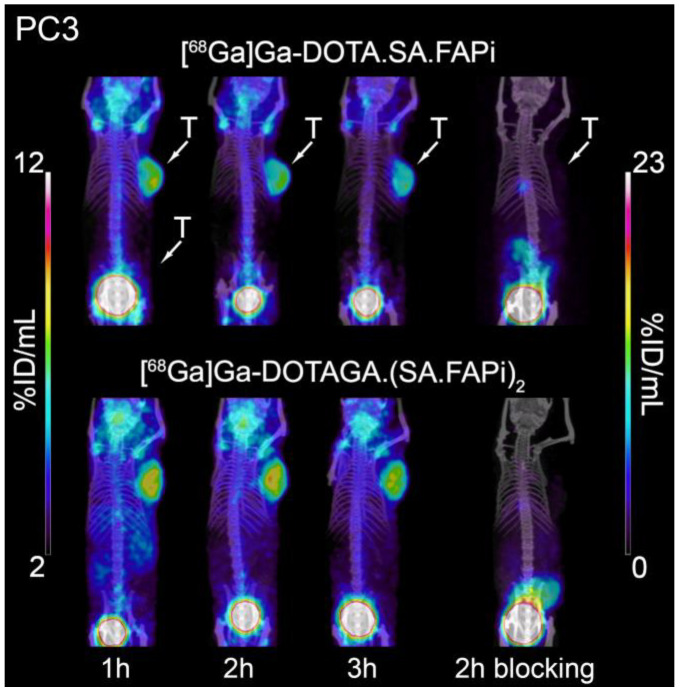
PET/CT images of PC3 tumor-bearing mice 1, 2, and 3 h after injection of [^68^Ga]Ga-DOTA.SA.FAPi and [^68^Ga]Ga-DOTAGA.(SA.FAPi)_2_ along with blocking studies at 2 h p.i.

**Figure 9:**
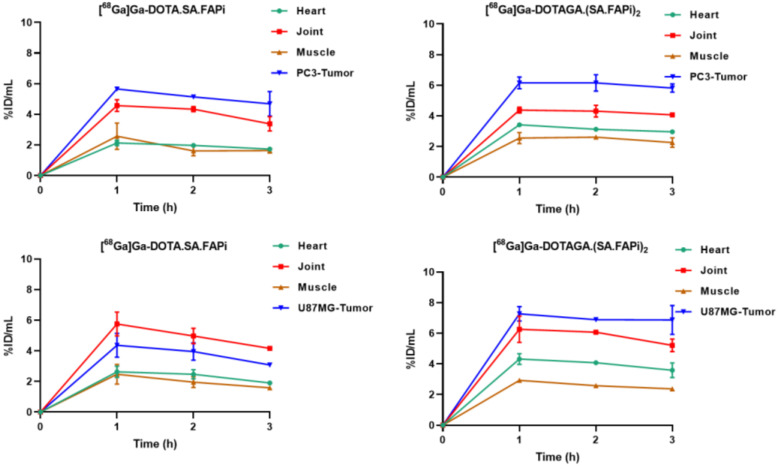
Quantitative analysis of the PET images of PC3 and U87MG tumor-bearing mice after injection of [^68^Ga]Ga-DOTA.SA.FAPi and [^68^Ga]Ga-DOTAGA.(SA.FAPi)_2_.

**Figure 10:**
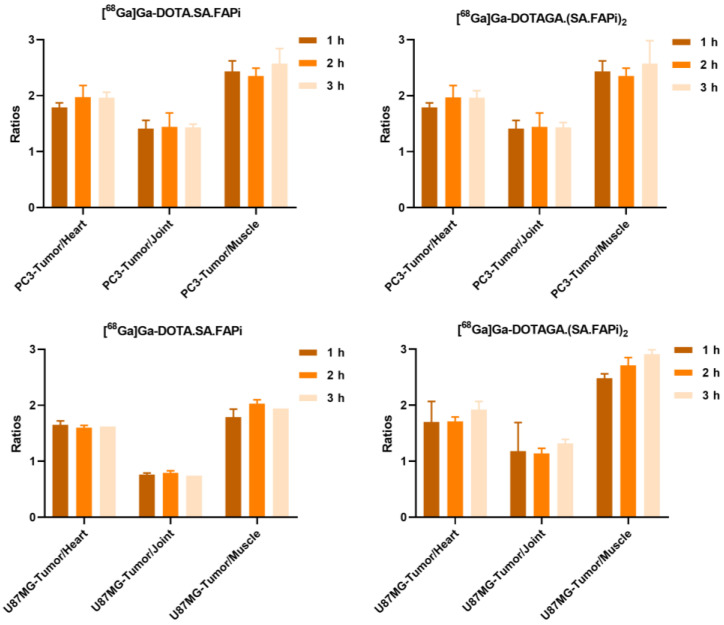
Tumor-to-tissue ratios generated from the quantification of the PET images of PC3 and U87MG tumor-bearing mice after the injection of [^68^Ga]Ga-DOTA.SA.FAPi and [^68^Ga]Ga-DOTAGA.(SA.FAPi)_2_, respectively.

Representative PET/SPECT/CT images of PC3 and U87MG tumor-bearing mice are depicted in Figure 7 and Figure 8. The U87MG and PC3 tumors are well delineated and the background, which is dominated by high blood pool, salivary glands and joints uptake, is higher for [^68^Ga]Ga-DOTAGA.(SA.FAPi)_2_ compared to [^68^Ga]Ga-DOTA.SA.FAPi. A quantitative analysis of the PET/CT images in terms of time-activity curves for the key organs and the corresponding tumor-to-tissue ratios is presented in Figure 9 and Figure 10, respectively. The PET-based quantification of the organ uptake aligns well with the ex vivo biodistribution data, revealing a higher blood pool (heart uptake in the PET images) for [^68^Ga]Ga-DOTAGA.(SA.FAPi)_2_ in comparison to [^68^Ga]Ga-DOTA.SA.FAPi. Furthermore, again in accordance with the biodistribution studies, PET imaging showed that the accumulated activity for [^68^Ga]Ga-DOTAGA.(SA.FAPi)_2_ in both the PC3 and U87MG tumors revealed slower washout over the course of 3 h compared to [^68^Ga]Ga-DOTA.SA.FAPi. Despite this, the tumor-to-tissue ratios of [^68^Ga]Ga-DOTAGA.(SA.FAPi)_2_ versus [^68^Ga]Ga-DOTA.SA.FAPi in both the PC3 and U87MG tumor models did not exhibit any significant differences.

**Figure 11:**
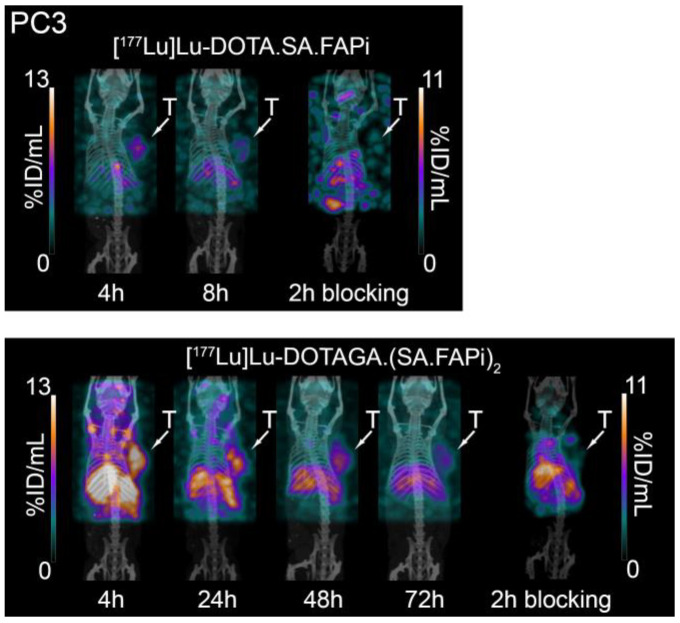
SPECT/CT images of PC3 tumor-bearing mice at several time points after injection of [^177^Lu]Lu-DOTA.SA.FAPi (**top**) and [^177^Lu]Lu-DOTAGA.(SA.FAPi)_2_ (**bottom**).

In line with the ex vivo biodistribution studies, the SPECT/CT images (Figure 11) clearly illustrate the superior tumor retention of [^177^Lu]Lu-DOTAGA.(SA.FAPi)_2_ compared to [^177^Lu]Lu-DOTA.SA.FAPi after their administration to PC3 tumor-bearing mice. The specificity of tumor uptake was confirmed through blocking experiments.

## 3. Discussion

**Figure 12:**
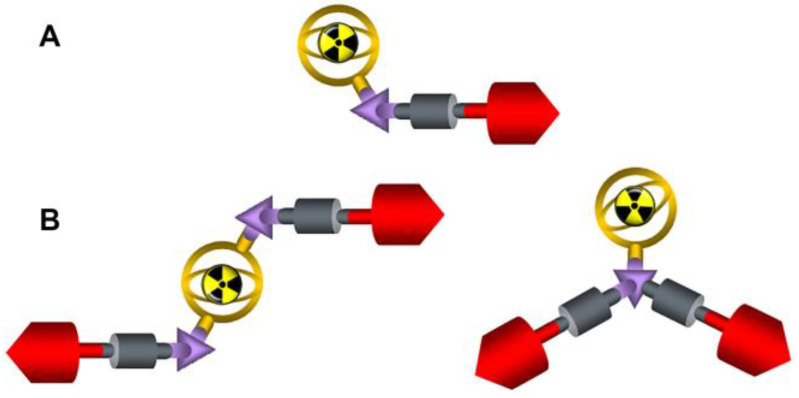
Schematic representation of monomeric (**A**) and dimeric (**B**) FAPi-based radiopharmaceuticals (red: targeting vectors, purple: central linker, grey: spacer, yellow: chelator-nuclide).

FAP imagining is a new approach in pan-cancer theranostics based on highly potent targeting vectors. In particular, the FAP inhibitor UAMC 1110 has been translated into gallium-68 and fluorine-18 labeled radiopharmaceuticals, which have been investigated to be equivalent or even superior to [^18^F]F-FDG [21,24,31,32,33,34,35,36]. The design of those compounds consists of a FAP inhibitor (FAPi) coupled to a spacer, a linker, a chelator and either the radiolabeled unit gallium-68 or fluorine-18. These structures are referred to as monomeric FAPi-based radiopharmaceuticals (Figure 12A). The initially great enthusiasm in the community to easily transform these diagnostic FAPi monomers into therapeutic analogs by simply exchanging the positron emitting gallium-68 with beta electron emitters such as yttrium-90 and lutetium-177 or the alpha emitting actinium-225 were somewhat tempered when a first in human study with a ^177^Lu-labeled FAP inhibitor did not meet the high expectations [20,37,38,39,40,41,42,43,44,45,46]. A major root cause for the disappointing therapeutic performance appeared to be the short residence times of the monomeric FAPi-based radiopharmaceuticals in the tumor microenvironment (TME), which was in the order of hours only, much shorter than the physical half-lives of the therapeutic radiometals used [27]. Fortunately, this problem was successfully addressed by developing homodimeric FAPI structures (Figure 12B) [4,6,10,11,12,13,14,17]. Compounds like DOTAGA.(SA.FAPI)_2_ or DOTAGA.Glu.(FAPI)_2_ remain in the lesions of various tumors over days and were found to substantially prolong overall and progression-free survival [20,22,23].

Although the strategy of increasing the residence time in the tumor by employing homodimeric instead of monomeric FAP inhibitors has proven successful, the underlying molecular basis and mechanism still remain elusive. In our current work, we aimed to gain a better understanding of the mechanisms governing the increase in the residence time at the oncological targets FAP or CAF when employing homodimeric instead of monomeric FAP inhibitors. We are confident that this exploration will not only enhance our understanding but also catalyze future advancements in the field. There are several concepts that may be relevant: (a) avidity versus affinity, (b) dual-binding mechanisms, (c) differences in membrane passage and internalization, (d) differences in externalization rates, and (e) active intracellular reactions by enzymes, i.e., trapping.

The present study first focused on the cellular aspects and investigated differences between monomeric and homodimeric FAP inhibitors labeled with trivalent radiometals, addressing binding affinity in terms of Kd values, membrane passage, and internalization and externalization rates. The systematic cell studies were then compared with the in vivo and ex vivo studies on tumor-bearing mice.

The chemical modification of the UAMC1110 lead structure with the SA linker and the chelator was well tolerated, as both DOTA.SA.FAPi and DOTAGA.(SA.FAPi)_2_, showed K_d_ values in the sub-nanomolar range. This compared well with data published by Moon et al. [12,13,14].

^177/nat^Lu-DOTA.SA.FAPi and ^177/nat^Lu-DOTAGA.(SA.FAPi)_2_ exhibited a somewhat reduced affinity when compared to ^68/nat^Ga-DOTA.SA.FAPi and ^68/nat^Ga-DOTAGA.(SA.FAPi)_2_, with this effect appearing to be more prominent for ^177/nat^Lu-DOTA.SA.FAPi. This may be explained by differences in terms of the charge and charge distribution arising after the coordination of gallium and lutetium in the chelator cavity [47].

Throughout all of the evaluated time points, internalization accounted for more than 95% of the total cellular activity. This phenomenon is influenced by several factors such as FAP expression, cell line characteristics, radioligand-FAP internalization mechanisms, affinity, and specificity. Furthermore, functional activation of FAP requires both dimerization and glycosylation, and given FAP’s short cytoplasmic domain, other receptors, such as integrins, may also act as intermediaries for FAP’s impact on the intracellular signaling and internalization rate [11,48]. Interestingly, in our study, the total internalized activity for the dimer was approximately 1.5 times lower than that of the monomer, indicating that different internalization mechanisms may be triggered upon the binding of monomers and dimers to FAP.

Considering the potential of the ^177^Lu-labeled radiotracers for therapeutic use, it is crucial to not only emphasize a strong binding affinity and high internalization rate but also to take into account the residence time of the radioactivity within cells. As part of our investigation, we conducted externalization studies, revealing notable distinctions between [^177^Lu]Lu-DOTA.SA.FAPi and [^177^Lu]Lu-DOTAGA.(SA.FAPi)_2_. For [^177^Lu]Lu-DOTA.SA.FAPi, after a 24 h period, only 7% of the total internalized radioactivity remained within the cells. In contrast, for [^177^Lu]Lu-DOTAGA.(SA.FAPi)_2_, a significant 48% of the total internalized radioactivity was retained in the cells. Undoubtedly, the ^177^Lu-labeled dimer exhibits a more favorable profile in the context of potential therapeutic possibilities. This corresponds to the observations made in patient studies [22].

Given the encouraging in vitro results and our prior success with FAP targeting of PC3 and U87MG tumor-bearing mice, we proceeded to in vivo evaluation using the same tumor models. While both [^68^Ga]Ga-DOTA.SA.FAPi and [^68^Ga]Ga-DOTAGA.(SA.FAPi)_2_ showed consistent results in terms of tumor uptake in both models, the dimer displayed slightly higher and more sustained absolute uptake, with no decline observed over time. In all cases, tumor uptake was specific, as demonstrated by the control group treated with the blocking agent. A considerably higher background uptake of [^68^Ga]Ga-DOTAGA.(SA.FAPi)_2_ in comparison to [^68^Ga]Ga-DOTA.SA.FAPi in both PC3 and U87MG tumor-bearing mice was observed. One possible explanation for this might be the elevated lipophilicity and the almost 2-fold higher serum protein binding of [^68^Ga]Ga-DOTAGA.(SA.FAPi)_2_ relative to [^68^Ga]Ga-DOTA.SA.FAPi. This could potentially be a limiting factor when employing [^68^Ga]Ga-DOTAGA.(SA.FAPi)_2_ for diagnostic applications.

An additional observation that warrants attention is the higher background uptake in the female U87MG compared to the male PC3 tumor-bearing mice for both radiotracers, possibly due to sex differences. Biodistribution studies of [^68^Ga]Ga-DOTA.SA.FAPi in healthy male and female mice supported the sex assumption. However, further research is required to validate these preliminary findings.

In view of their potential therapeutic application, a direct in vivo comparison of the ^177^Lu-labeled radiotracers was conducted in PC3 tumor-bearing mice. Even though both radiotracers exhibited early and relatively high tumor uptake, they demonstrated distinct clearance rates in line with our externalization data. [^177^Lu]Lu-DOTAGA.(SA.FAPi)_2_ maintained a significant tumor uptake even after 96 h, whereas [^177^Lu]Lu-DOTA.SA.FAPi was nearly completely washed out already at 48 h p.i. The elevated liver uptake observed for [^177^Lu]Lu-DOTAGA.(SA.FAPi)_2_ across all examined time points could be attributed to its increased lipophilicity compared to [^177^Lu]Lu-DOTA.SA.FAPi. To achieve the appropriate balance and consequently the desired in vivo behavior, additional chemical modifications on the dimeric precursor’s structure may be required [10].

Both ^68^Ga- and ^177^Lu-labeled radiotracers demonstrated elevated uptake in non-target organs including muscles, lungs, pancreas, salivary glands, and joints, a phenomenon also noted in clinical settings [3,7,8,17,19,20,21,22,23,26,29,49]. The development of these organs is, in part, influenced by the activity of fibroblasts and this might provide an explanation for the off-target uptake. The non-target organs uptake was lower for the monomer compared to the dimer and also for the male compared to the female mice. Additionally, the monomer exhibited a higher rate of washout over time (Figure S3).

The utilization of diverse tumor models from individual groups precludes a straightforward comparison between [^177^Lu]Lu-DOTAGA.(SA.FAPi)_2_ and the previously reported ^177^Lu-labeled dimeric FAPi-based radiopharmaceuticals. Nevertheless, a consistent pattern was observed across all, indicating an enhanced accumulation and extended retention time of the dimers in tumors compared to monomers [6,11,15,17]. This highlights the promising therapeutic potential of the dimeric concept.

The preclinical findings presented here are consistent with an initial dosimetry study involving a limited patient cohort where [^177^Lu]Lu-DOTAGA.(SA.FAPi)_2_ (10 patients; breast cancer (four), thyroid cancer (five), paraganglioma (one)) exhibited significantly prolonged retention in tumors compared to [^177^Lu]Lu-DOTA.SA.FAPi (three patients; breast cancer) [20,21]. The median absorbed tumor doses in the group of patients treated with [^177^Lu]Lu-DOTA.SA.FAPi were found to be 0.603 (IQR: 0.230–1.810) Gy/GBq per cycle of treatment, while the respective value for [^177^Lu]Lu-DOTAGA.(SA.FAPi)_2_ was 6.70 (IQR: 3.40–49) Gy/GBq. Although subsequent accumulations of [^177^Lu]Lu-DOTAGA.(SA.FAPi)_2_ in the bone marrow and kidneys exceeded those observed with [^177^Lu]Lu-DOTA.SA.FAPi, they are still well-tolerated and are also in line with the tumor/organ-absorbed doses of [^177^Lu]Lu-DOTATATE and [^177^Lu]Lu-PSMA-617 in neuroendocrine tumors and prostate cancer, respectively [50,51,52]. Further clinical studies on patients with aggressive medullary thyroid carcinoma [23] and breast cancer [49] demonstrated the therapeutic potential of [^177^Lu]Lu-DOTAGA.(SA.FAPi)_2_, which may pave the way for theranostic interventions in end-stage cancer patients. The already existing dosimetry data on FAP targeted radionuclide therapy indicate that the absorbed tumor dose varies between 0.62 ± 0.55, 2.81 ± 1.25, 3.0 ± 2.7 and 6.70 Gy/GBq for [^177^Lu]Lu-FAPI-04, [^90^Y]Y-FAPI-46, [^177^Lu]Lu-FAP-2286 and [^177^Lu]Lu-DOTAGA.(SA.FAPi)_2_, respectively [20,23,25,45,53,54,55], supporting the success of the dimeric approach to prolong the retention time of the FAP tracers in tumors.

## 4. Materials and Methods

### 4.1. Radiolabeling/Quality Control of the Radiotracers/Stability

^68^Ga-labelings were performed using the Modular-Lab PharmTracer module (Eckert & Ziegler Berlin, Germany), and ^177^Lu-labelings were performed manually (Supplemental Data). Radiochemical purity and stability for 4 h for the ^68^Ga-labelled and 48 h for the ^177^Lu-labelled radiotracers were determined by reversed-phase high performance liquid chromatography (RP-HPLC) and radio thin-layer chromatography (radio-TLC).

### 4.2. Lipophilicity/Protein Binding Studies

The lipophilicity (LogD_octanol/PBS_) and protein binding of the four radiotracers in human serum were determined as described in the Supplemental Data.

### 4.3. Cell Lines

PC3 (Cell Lines Service GmbH (CLS, Eppelheim, Germany), U87MG (American Type Culture Collection (ATCC, Manassas, VA, USA) and CAF (American Type Culture Collection (ATCC, Manassas, VA, USA) cells have been used. Cultivation conditions, materials and further details are described in the Supplemental Data (Gibco BRL, Life Technologies (Grand Island, NY, USA).

### 4.4. Saturation Binding/Internalization/Externalization Studies

For saturation studies, CAFs were incubated with increasing concentrations of the radiotracers (0.1–10 nM). For internalization studies, approximately 2.5 pmol of the radiotracers were added to CAFs followed by incubation for 15, 30, 60, 90, 120, 180 and 240 min for the ^68^Ga-labeled or 30, 60, 120, 240 and 360 min for the ^177^Lu-labeled radiotracers at 37 °C, 5% CO_2_. For the externalization studies, approximately 2.5 pmol of the ^177^Lu-labeled radiotracers were added to CAFs followed by incubation for 2 h at 37 °C, 5% CO_2_. Afterward, the amount of the externalized activity from the CAFs was determined at 0, 10, 20, 30, 60, 120, 240, and 1440 min (Supplemental Data).

### 4.5. Animal Models

**U87MG and PC3 tumor-bearing mice**: Female athymic BALB/c (6 weeks/16–20 g) and male athymic BALB/c nude mice (6 weeks/20–25 g) were implanted with U87MG or PC3 cells (5 × 10^6^/100 µL PBS) into their right shoulder, respectively. The animals were used for biodistribution and PET/SPECT/CT imaging studies (Animal License No: BE98/2021).

### 4.6. Biodistribution Studies

#### 4.6.1. [^68^Ga]Ga-DOTA.SA.FAPi and [^68^Ga]Ga-DOTAGA.(SA.FAPi)_2_

Ten pmol (0.08–0.1 MBq) of the radiotracers in 100 µL of NaCl 0.9% were injected intravenously into the tail vein of U87MG and PC3 tumor-bearing mice and biodistribution studies were conducted at 1, 2, and 3 h after injection. To demonstrate the specificity of binding, the mice were co-injected with 10 pmol of each radiotracer along with 20 nmol of UAMC1110 (total injected volume: 100 µL) and the biodistribution studies were performed 2 h p.i.

Two groups of healthy mice (males and females; *n* = 4/group) were injected with 10 pmol of [^68^Ga]Ga-DOTA.SA.FAPi (0.08–0.1 MBq) in 100 µL of NaCl 0.9% and biodistribution studies were conducted at 1 h p.i. (Supplemental Data).

#### 4.6.2. [^177^Lu]Lu-DOTA.SA.FAPi and [^177^Lu]Lu-DOTAGA.(SA.FAPi)_2_

Ten pmol (0.06–0.08 MBq) of the radiotracers in 100 µL of NaCl 0.9% were injected intravenously into the tail vein of PC3 tumor-bearing mice. Biodistribution studies were conducted at 1, 4, 24, and 48 h after the injection of [^177^Lu]Lu-DOTA.SA.FAPi and at 4, 24, 48, 72, and 96 h after the injection of [^177^Lu]Lu-DOTAGA.(SA.FAPi)_2_ (Supplemental Data).

### 4.7. Small-Animal PET/SPECT/CT Imaging

PET images were obtained upon injection of 200 pmol of the ^68^Ga-labeled radiotracers (1.0–1.6 MBq/100 μL) in U87MG and PC3 tumor-bearing mice. SPECT images were obtained upon injection of 1000 pmol (~11 MBq/100 μL) of [^177^Lu]Lu-DOTA.SA.FAPi and [^177^Lu]Lu-DOTAGA.(SA.FAPi)_2_ in PC3 tumor-bearing mice (Supplemental Data).

### 4.8. Statistical Analysis

Data are expressed as mean ± standard deviation (mean ± SD). Prism 8 software (GraphPad Software 8) was used to determine the statistical significance at the 95% confidence level, with a *p*-value of less than 0.05 being considered significantly different.

## 5. Conclusions

[^68^Ga]Ga-DOTA.SA.FAPi exhibited highly advantageous characteristics for imaging. These qualities encompass tumor-to-background ratios that highlight its excellent utility in diagnostic applications for a wide range of cancer types. The present comprehensive preclinical evaluation of [^177^Lu]Lu-DOTAGA.(SA.FAPi)_2_ revealed substantial potential for effective radionuclide-based targeted therapy against FAP-positive tumors, supporting the current clinical data. Although this is not a “true” radiotheranostic pair, the optimal synergy of monomers for imaging and dimers for therapy could be a pivotal factor in the successful management of FAP-positive tumors. Further clinical trials are required to evaluate the efficacy and safety of various radiotheranostic combinations, providing hope for improved cancer care.

## Figures and Tables

**Table 1:** LogD_octanol/PBS-pH7.4_ and Percentage of Human Serum Protein Bound Activity.

	DOTA.SA.FAPi		DOTAGA.(SA.FAPi)_2_	
	[^68^Ga]Ga-	[^177^Lu]Lu-	[^68^Ga]Ga-	[^177^Lu]Lu-
**Lipophilicity**	−3.38 ± 0.03	−2.86 ± 0.06	−1.83 ± 0.02	−1.71 ± 0.03
**Protein Binding**	10.6 ± 3.9%	9.9 ± 4.4%	18 ± 1.1%	25.3 ± 0.8%

## Data Availability

All data are contained within the article or Supplementary Materials.

## Supplementary Materials

The following supporting information can be downloaded at: https://www.mdpi.com/article/10.3390/molecules29133093/s1, Figure S1: Schematic representations of DOTA.SA.FAPi (monomer) and DOTAGA.(SA.FAPi)_2_ (dimer); Figure S2: HPLC profile of [^68^Ga]Ga-DOTA.SA.FAPi; Figure S3: HPLC profile of [^68^Ga]Ga-DOTAGA.(SA.FAPi)_2_; Figure S4: HPLC profile of [^177^Lu]Lu-DOTA.SA.FAPi; Figure S5: HPLC profile of [^177^Lu]Lu-DOTAGA.(SA.FAPi)_2_; Figure S6: Representative TLC profiles in (a) Sodium Citrate and (b) Ammonium Acetate:MeOH of [^68^Ga]Ga-DOTA.SA.FAPi; Figure S7: Saturation binding study on CAF cells, using increasing concentrations of ^68/nat^Ga-DOTA.SA.FAPi (^68/nat^Ga-monomer) and ^68/nat^Ga-DOTAGA.(SA.FAPi)_2_ (^68/nat^Ga-dimer) (0.1 to 10 nM). Dissociation constant (K_d_) and maximum number of binding sites (B_max_) were calculated from nonlinear regression analysis using GraphPad Prism 8; Figure S8: Total surface bound and non-specific cell bound uptake after the incubation of CAFs with [^68^Ga]Ga-DOTA.SA.FAPi, [^68^Ga]Ga-DOTAGA.(SA.FAPi)_2_, [^177^Lu]Lu-DOTA.SA.FAPi and [^177^Lu]Lu-DOTAGA.(SA.FAPi)_2_ within 4 and 6 h at 37 °C. The uptakes are expressed as percentage of the total applied radioactivity. Nonspecific binding was determined in the presence of 1 μM UAMC1110; Figure S9: Representative slices (thickness 0.25 mm) of PET/CT images of PC3 (male) and U87MG (female) xenografts upon injection of [^68^Ga]Ga-DOTA.SA.FAPi and [^68^Ga]Ga-DOTAGA.(SA.FAPi)_2_ at 1, 2 and 3 h after injection (SL: salivary glands); Table S1: Biodistribution data and tissue radioactivity ratios of [^68^Ga]Ga-DOTA.SA.FAPi and [^68^Ga]Ga-DOTAGA.(SA.FAPi)_2_ on PC3 xenographs; Table S2: Biodistribution data and tissue radioactivity ratios of [^68^Ga]Ga-DOTA.SA.FAPi and [^68^Ga]Ga-DOTAGA.(SA.FAPi)_2_ on U87MG xenographs; Table S3: Biodistribution Data of [^68^Ga]Ga-DOTA.SA.FAPi on Healthy Male and Female Mice at 1 h p.i.; Table S4: Biodistribution data and tissue radioactivity ratios of [^177^Lu]Lu-DOTA.SA.FAPi and [^177^Lu]Lu-DOTAGA.(SA.FAPi)_2_ on PC3 xenographs.
