# Supplementary material for: Tailoring Fibroblast-Activation Protein Targeting for Theranostics: A Comparative Preclinical Evaluation of the 68Ga- and 177Lu-Labeled Monomeric and Dimeric Fibroblast-Activation Protein Inhibitors DOTA.SA.FAPi and DOTAGA.(SA.FAPi)2
Source: Molecules. 2024 Jun 28;29(13):3093. doi: 10.3390/molecules29133093 (PMC11243320; doi:10.3390/molecules29133093)
Supplement: Supplementary file 1 [file molecules-29-03093-s001.zip › molecules-3039639-supplementary.pdf]

molecules

Supplemental Data

Tailoring Fibroblast-Activation Protein Targeting for Theranostics: A Comparative Preclinical Evaluation of the ^{68}Ga- and ^{177}Lu-Labeled Monomeric and Dimeric Fibroblast-Activation Protein Inhibitors DOTA.SA.FAPi and DOTAGA.(SA.FAPi)$_2$

Tilman Läppchen [1], Adrianna Bilinska [1], Eirinaios Pilatis [1], Elena Menéndez [1], Surachet Imlimthan [1], Euy Sung Moon [2], Ali Afshar-Oromieh [1], Frank Rösch [2], Axel Rominger [1] and Eleni Gourni [1,*]

[1] Department of Nuclear Medicine, Inselspital, Bern University Hospital, city, post code, Switzerland; tilman.laeppchen@insel.ch (T.L.); adrianna.bilinska@extern.insel.ch (A.B.); eirinaios.pilatis@insel.ch (E.P.); elena.menendez@insel.ch (E.M.); surachet.imlimthan@helsinki.fi (S.I.); ali.afshar@insel.ch (A.A.-O.); axel.rominger@insel.ch (A.R.)
[2] Department of Chemistry – TRIGA site, Johannes Gutenberg-University Mainz, city, post code, Germany; esmoon92@googlemail.com (E.S.M.); froesch@uni-mainz.de (F.R.)
* Correspondence: eleni.gourni@insel.ch; Tel.: +41316640507

Materials and Methods

Reagents and Instrumentation

All reagents were of the best grade available and were purchased from common suppliers. All culture reagents were from Gibco BRL, Life Technologies (Grand Island, NY). The cancer associated prostate fibroblast cell line (hTERT PF179T, ATCC CRL-3290, Lot Number 0303) and the human glioblastoma and the epithelial prostate cancer cell line (U87MG, ATCC HTB-14, Lot number 4095) were acquired from American Type Culture Collection (ATCC, Manassas, Virginia, USA). The human prostate adenocarcinoma cell line PC3 (CLS-300312, Lot number 816SF) was obtained from Cell Lines Service GmbH (CLS, Eppelheim, Germany). The human serum was commercially available from Sigma (H4522). [^{177}Lu]LuCl$_3$ in 0.04 M HCl was obtained from ITM, Pharma Solution GmbH (Garching/Munich, Germany) and DSD-Pharma GmbH (Purkersdorf near Vienna, Austria). The molar activity is ≥ 3000 GBq/mg for both n.c.a. products (Lu-177). The GalliaPharm® Ge-68/Ga-68 generator was available from Eckert & Ziegler (Berlin, Germany). The quality control of the precursor and the radiotracers was performed by analytical reverse-phase high performance liquid chromatography (RP-HPLC) on an analytical Nucleosil 120-5 column C18 (250 × 4.5 mm) applying a linear gradient of 15–90% solvent B in 30 min at a flow rate of 1 mL/min. (solvent A, 0.1% trifluoroacetic acid (TFA)/water (H_2O); solvent B, 0.1%TFA/acetonitrile (ACN). Ultraviolet detection was performed using an Agilent detector at 214 nm. For radioactivity measurement, a Na(TI) well-type scintillation Gina star was used. The radiotracer solutions for the experiments were prepared by dilution with 0.9% NaCl (Bichsel AG, Interlaken, Switzerland).

Quantitative γ-counting was performed with a COBRA II γ-system well counter from Packard Instruments (USA). For the μPET/CT studies, a dedicated micro-PET/SPECT/CT scanner (Albira Si; Bruker Biospin, Ettlingen, Germany) was used.

All experiments were carried out two times in triplicate.

Mice were purchased from Charles River Laboratories (Domain des Oncins, France) and the pentobarbital natrium (150 mg/kg) was from Streuli Pharma SA (Uznach, Switzerland).

Radiochemistry/Quality Control of the Radiotracers

Radiolabeling with Gallium-68

[^{68}Ga]Ga-DOTA.SA.FAPi and [^{68}Ga]Ga-DOTAGA.(SA.FAPi)$_2$ were prepared within 5 min, using the Modular-Lab PharmTracer module by Eckert & Ziegler (Berlin, Germany). The radiolabeling performance of DOTA.SA.FAPi and DOTAGA.(SA.FAPi)$_2$ was assessed at pH 4.0 (0.2 M sodium acetate buffer or 1.45 M HEPES buffer for DOTA.SA.FAPi and DOTAGA.(SA.FAPi)$_2$, respectively), at 90 °C using, in each case, the

minimum conjugate amount. Briefly, the Ge-68/Ga-68 generator was eluted with 5 mL HCl 0.1 N and the eluate (~300 MBq) was loaded onto a cation exchange column (Strata-XC, Phenomenex). Gallium-68 was eluted with 700 μL of a mixture of 5.5 M NaCl / 0.1 M HCl (prepared by mixing 0.530 mL of ultrapure 30% aq. HCl and 49.5 mL of 5.5M NaCl) directly into a vial containing 400 μL of 1.8 M sodium acetate buffer (pH 4.3) for DOTA.SA.FAPi or 2 mL 1.45 M HEPES (pH 4.1) for DOTAGA.(SA.FAPi)$_2$, 2 mL H_2O (only in case of DOTA.SA.FAPi), 200 μL of EtOH, and 20 μg (approximately 20 nmol) of the conjugate, followed by Sep-Pak C-18 purification to remove uncomplexed gallium-68. The purified [^{68}Ga]Ga-DOTA.SA.FAPi and [^{68}Ga]Ga-DO,TAGA.(SA.FAPi)$_2$ were eluted in 2.7 mL of a solution of 12% EtOH in PBS.

The quality control of [^{68}Ga]Ga-DOTA.SA.FAPi and [^{68}Ga]Ga-DOTAGA.(SA.FAPi)$_2$ was performed by radio-HPLC as described in the reagents and instrumentation. The presence of free gallium-68 and ^{68}Ga-labelled colloid in the [^{68}Ga]Ga-DOTA.SA.FAPi and [^{68}Ga]Ga-DOTAGA.(SA.FAPi)$_2$ preparations was quantified by radio thin layer chromatography (Radio-TLC) using ITLC-SG-plates (glass microfiber chromatography paper impregnated with silica gel) and two different mobile phase eluents: a) 0.1 M Na-citrate; b) MeOH/1 M ammonium acetate (1/1, v/v).

Radiolabeling with Lutetium-177

[^{177}Lu]Lu-DOTA.SA.FAPi and [^{177}Lu]Lu-DOTAGA.(SA.FAPi)$_2$ were prepared by dissolving 5-13 μg (approximately 5-12 nmol) of DOTA.SA.FAPi and DOTAGA.(SA.FAPi)$_2$, respectively, in 250 μL ammonium acetate buffer (0.5 M, pH 5.4) or HEPES buffer (1 M, pH 5.5), followed by incubation with [^{177}Lu]Lu^{3+} (30 - 130 MBq) at 90 °C for 30 min. Both products were used without any further purification step.

The quality control of [^{177}Lu]Lu-DOTA.SA.FAPi and [^{177}Lu]Lu-DOTAGA.(SA.FAPi)$_2$ was performed by radio-HPLC and radio thin layer chromatography (radio-TLC) as described in the reagents and instrumentation.

After the labeling with gallium-68 or lutetium-177 and the quality control of the generated radiotracers, one equivalent of either $^{nat}Ga(NO_3)_3$ or $^{nat}LuCl_3{\cdot}6H_2O$ was added to the relevant radiolabeling solutions. The final solutions were incubated at 90 °C for 30 min to obtain the structurally characterized homogeneous ligands, which were used for the saturation binding studies. The homogeneity was determined by HPLC, showing one peak.

Lipophilicity/Protein Binding Studies and Metabolic Stability in Human Serum

The lipophilicity ($LogD_{Octanol/PBS}$, pH 7.4) was estimated by the "shake–flask" method: the labelled conjugate (20 pmol; 0.3 MBq) was added to a 1:1 mixture of 1-octanol (500 μL) and PBS (500 μL, pH 7.4). The mixture was intermittently vortexed for 1 h to reach the equilibrium and then centrifuged (3000 rpm) for 10 min. From each phase, an aliquot (50 μL) was pipetted out and measured in a gamma counter. Each measurement was repeated five times. Care was taken to avoid cross-contamination between the phases. The partition coefficient was calculated as the average log ratio of the radioactivity in the organic fraction and the PBS fraction.

The ^{68}Ga- and ^{177}Lu-labelled radiotracers (20 pmol; 0.3 MBq) were incubated with commercially available human serum (0.5 mL) at 37 °C for 30 min. When the incubation period was completed, the proteins were precipitated with a solution of 1 mL MeOH/ACN (1:1). Centrifugation (10 min, 9660g) was performed for the separation of the proteins. After careful separation of the two phases, the respective activities were measured in a gamma counter, followed by determination of the percentage of the radiotracer that bound to the serum proteins.

Cell Lines

The human prostate adenocarcinoma cell line PC3 was cultured in Dulbecco's Modified Eagle Medium (DMEM) with low glucose (1 g/L) combined with F-12 Nutrient Mix with GlutaMAX™-I (1:1 mixture ratio). The human glioblastoma cell line U87MG was cultured in Dulbecco's Modified Eagle Medium (DMEM) with low glucose (1 g/L) supplemented with NEAA 1% and sodium pyruvate 1 mM. The cancer-associated prostate fibroblast cell line CAF was cultured in the same conditions with Eagle's Minimal Essential Medium (EMEM) supplemented with sodium bicarbonate 1500 mg/L and puromycin 1 μg/mL. In each case, the medium was supplemented with 10% fetal bovine serum (FBS), penicillin (100 U/mL) and streptomycin (100 μg/mL). All of the cell lines were cultured at 37 °C and 5% CO_2.

Saturation Binding Studies

For the receptor saturation analysis, the CAF cell line overexpressing FAP was seeded at a density of 0.8-1 million cells per well in 6-well plates pre-coated with BME (Cultrex Reduced Growth Factor Basement Membrane Extract, RGF BME, R&D System, Minneapolis, USA) to enhance cell attachment. The cells were incubated overnight with medium (EMEM containing 10% FBS, 100 U/mL penicillin and 100 μg/mL streptomycin). The next day, the medium was removed and the cells were incubated for 30 min at 37 °C with 0.8 mL of fresh medium. Afterward, the plates were placed on ice for 30 min followed by incubation with increasing concentrations of $^{68/nat}$Ga-labeled and $^{177/nat}$Lu-labeled DOTA.SA.FAPi and DOTAGA.(SA.FAPi)$_2$ (0.1-10 nM final concentration in the wells; 100 μL of radioligand solution and 100 μL of PBS pH 7.4 were added to the cells with 0.8 mL medium in the well). After the addition of the radioligands, the cells were incubated for 120 min at 4 °C. Non-specific binding was determined in the presence of UAMC1110 at a final concentration of 1 μM. After the completion of the incubation, the cells were washed twice with ice-cold PBS, followed by solubilization with 1 N NaOH. The cell-associated radioactivity was measured using a gamma counter. Specific binding was plotted against the total molar concentration of the added radiotracer. The K_d values and the concentration of the radiotracer required to saturate the receptors (B_{max}) were determined by nonlinear regression using GraphPad (Prism 8 Graph Pad Software, San Diego, CA). For all of the cell studies the values are normalized for 1x10^6 cells per well and all data are from two independent experiments with triplicates in each experiment.

Internalization Studies

For the internalization experiments, CAF cells were seeded into 6-well plates pre-coated with BME and treated as described in the saturation binding studies. On the day of the experiment, approximately 2.5 pmol (100 μL) of the radiotracer was added to the medium (total volume 1.5 mL) and the cells were incubated (in triplicates) for 15, 30, 60, 90, 120, 180 and 240 min at 37 °C, 5% CO_2 for the ^{68}Ga-labeled tracers and for 0.5, 1, 2, 4 and 6 h at 37 °C, 5% CO_2 for the ^{177}Lu-labeled tracers. To identify the nonspecific membrane binding and internalization, an excess of UAMC1110 (final concentration 1 μM) was added to the selected wells. At each time point, the internalization was stopped by putting the plates on ice, removing the medium, and washing the cells twice with ice-cold PBS. To remove the receptor-bound radioligand, an acid wash was carried out twice with a 0.1 M glycine buffer pH 2.8 for 5 min on ice. Finally, the cells were solubilized with 1 N NaOH. The radioactivity of the culture medium, the receptor bound, and the internalized fractions were measured in a γ-counter.

Externalization Studies

The externalization rate of [^{177}Lu]Lu-DOTA.SA.FAPi and [^{177}Lu]Lu-DOTAGA.(SA.FAPi)$_2$ was studied after CAFs were incubated for 2 h with 2.5 pmol/well of each ^{177}Lu-lebeled radioligand. After the completion of the 2 h of incubation, the medium was removed and the CAF cells were washed with 1 ml of cold PBS (×2). The cells were then exposed to an acid wash with glycine buffer, as described above, to dissociate the cell surface-bound radioligand, and the fractions were collected. Fresh medium (1 mL/well) was added to the cells and were incubated at 37 °C. At different time points (10, 20, 30, 60, 120, 240 and 1440 min) the external medium was removed (followed by two washing steps with cold PBS). Finally, the cells were solubilized with 1 N NaOH. The radioactivity of the culture medium, the receptor bound, the internalized and the externalized fractions were measured in a γ-counter. The externalized fraction of radioactivity was expressed as the percentage of the total internalized amount.

Biodistribution Studies

[^{68}Ga]Ga-DOTA.SA.FAPi and [^{68}Ga]Ga-DOTAGA.(SA.FAPi)$_2$

Ten pmol (0.08–0.1 MBq) of [^{68}Ga]Ga-monomer and [^{68}Ga]Ga-dimer in 100 μL of NaCl 0.9% were injected intravenously into the tail vein of U87MG or PC3 tumor bearing mice. The PC3 tumor-bearing mice were used between the 15th and 17th day after the xenograft implantation and the size was 281 ± 75 mm^3. The U87MG tumor-bearing mice were used between the 7th and 9th day after the implantation and the size was 210 ± 34 mm^3. Animals were terminally anesthetized by intraperitoneal injection of an overdose of pentobarbital natrium (150 mg/kg; Streuli Pharma SA, Uznach, Switzerland) at 1, 2 and 3 h after injection of the radiotracers. The organs of interest were dissected and weighed, and the radioactivity in the tissue samples

was counted in a γ-counter. Biodistribution data are given as the percent of injected activity per gram of tissue (% IA/g) and as means ± SD (n = 4).

To demonstrate the specificity of binding, U87MG or PC3 xenografts were co-injected with 10 pmol of each radiotracer along with 20 nmol of UAMC1110 (total injected volume: 100 μL) and biodistribution studies were performed 2 h after injection.

[^{177}Lu]Lu-DOTA.SA.FAPi and [^{177}Lu]Lu-DOTAGA.(SA.FAPi)$_2$

Ten pmol (0.06-0.08 MBq) of [^{177}Lu]Lu-monomer and [^{177}Lu]Lu-dimer in 100 μL of NaCl 0.9% were injected intravenously into the tail vein of PC3 tumor-bearing mice. The PC3 tumor-bearing mice were used between the 15th and 17th day after the xenograft implantation and its size was 281 ± 75 mm^3. Animals were terminally anesthetized by intraperitoneal injection of an overdose of pentobarbital natrium (150 mg/kg; Streuli Pharma SA, Uznach, Switzerland) 1, 4, 24 and 48 h after the injection of [^{177}Lu]Lu-DOTA.SA.FAPi and at 4, 24, 48, 72 and 96 h after the injection of [^{177}Lu]Lu-DOTAGA.(SA.FAPi)$_2$. The organs of interest were dissected and weighed, and the radioactivity in the tissue samples was counted in a γ-counter. The biodistribution data are given as the percent of injected activity per gram of tissue (% IA/g) and as means ± SD (n = 4).

After the biodistribution studies, the tumors were checked for their vascularization and if they possessed blood pools or if they were necrotic. Both tumor types were "clean" without extended vascularization; there was no blood pooled in the center of the tumors, and we also did not observe any necrotic signs.

Small Animal Imaging Studies

PET/CT Studies

PET images were obtained after injection of 200 pmol of [^{68}Ga]Ga-DOTA.SA.FAPi and [^{68}Ga]Ga-DOTAGA.(SA.FAPi)$_2$ (1.0 – 1.6 MBq/100 μL) into U87MG or PC3 xenografts at 1, 2 and 3 h after injection. Static imaging was acquired for a time period between 10 and 45 min at 1, 2 and 3 h post injection, respectively. To visualize the extent of FAP-specific tumor uptake, PET/CT blocking studies were performed as described above, and static scans were obtained for 30 min 2 h after the injection of the radiotracer along with the blocking agent (20 nmol).

PET images were corrected for gallium-68 decay and reconstructed with 12 iterations of the maximum a posteriori (MAP) algorithm using a voxel size of 0.25 mm. No correction was applied for attenuation. In addition, a point spread function iterative deconvolution was applied. The images were normalized, filtered using a Gaussian 3D algorithm with a 1.3 mm isotropic kernel and generated using PMOD software. The CT was carried out using step and shoot mode and employed 45 kVp and 400 uA as settings. The images were reconstructed using a filtered back projection algorithm. The color scale was set from 2 to 12 % IA/g to allow for qualitative comparisons among the images.

SPECT/CT Studies

SPECT images were obtained upon injection of 1000 pmol (~11 MBq/100 μL) [^{177}Lu]Lu-DOTA.SA.FAPi and [^{177}Lu]Lu-DOTAGA.(SA.FAPi)$_2$ into the PC3 xenografts at 4 and 24 h p.i. in the case of [^{177}Lu]Lu-DOTA.SA.FAPi and at 4, 24, 48, 72 h p.i. in the case of [^{177}Lu]Lu-DOTAGA.(SA.FAPi)$_2$. To visualize the extent of FAP-specific tumor uptake, SPECT/CT blocking studies were performed as described above, and static scans were obtained for 45 min 4 h after the injection of the radiotracers along with the blocking agent (20 nmol).

SPECT was acquired using a 208 keV ± 20% energy window and a Cerrobase (Bi-Pb alloy) 30 mm thick collimator with a pinhole 22 mm thick tungsten lens. Raw SPECT data were reconstructed using the Ordered Subset Expectation Maximization (OSEM) algorithm for 3 iterations of 5 subsets and a voxel size of 0.25mm. The reconstructed data were corrected for Lutetium-177 decay, normalized, and filtered using a Gaussian 3D algorithm with a 2 mm isotropic kernel and generated using PMOD software. The CT was carried out using step-and-shoot mode, employing 45 kVp and 400 μA as settings. The images were reconstructed using the Filtered Back Projection (FBP) algorithm and a voxel size of 0.25 mm. The color scale of the SPECT images was set as 0 to 13% IA/mL allowing for qualitative comparisons among the images.

Results

DOTA.SA.FAPi (monomer) and DOTAGA.(SA.FAPi)2 (dimer)

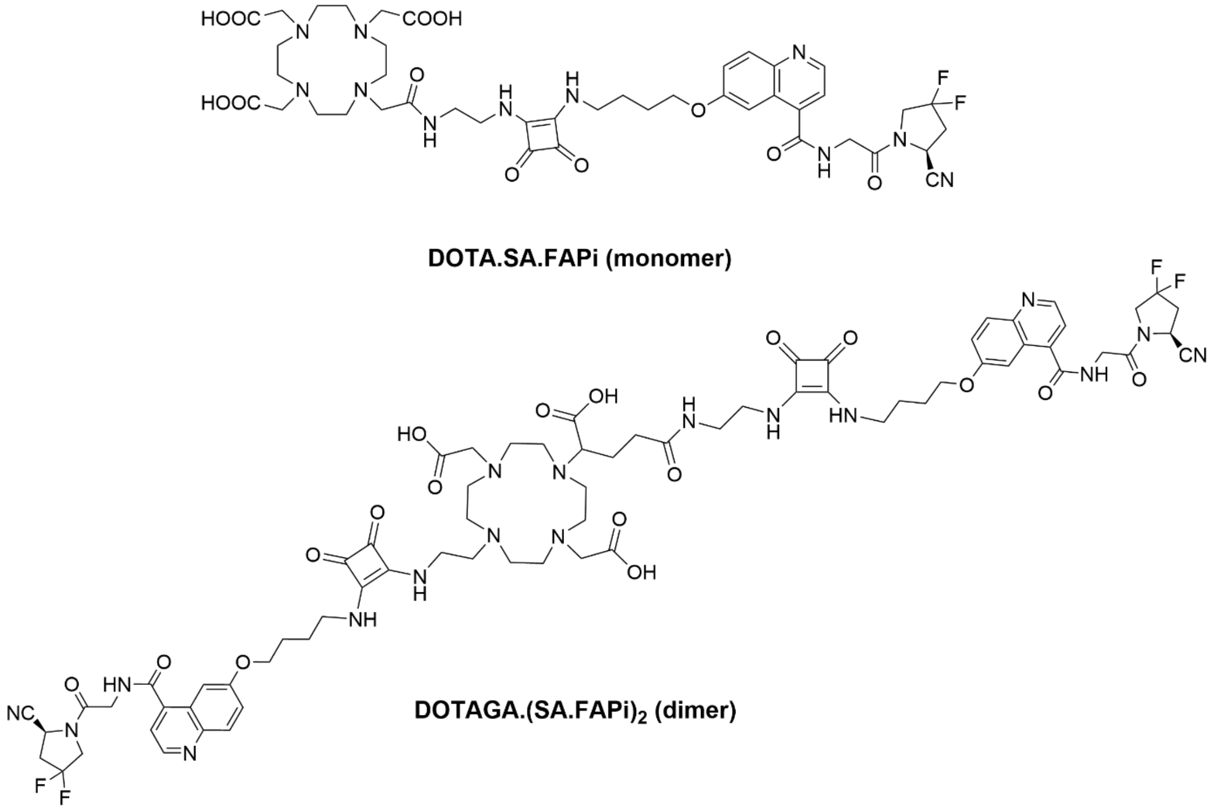

Figure S1. Schematic representations of DOTA.SA.FAPi (monomer) and DOTAGA.(SA.FAPi)2 (dimer).

Quality Control of the Radiotracer/Stability

Based on the amount of the precursor that was used for the radiolabeling and assuming we lose about 20% during the labeling, the apparent molar activity (A_m) was 8.5 ± 0.8 and 12.4 ± 4.4 GBq/µmol for [^{68}Ga]Ga-DOTA.SA.FAPi and [^{68}Ga]Ga-DOTAGA.(SA.FAPi)2, respectively (not decay corrected).

With regard to the detection of the formation of colloids by ITLC, using the first radio-TLC eluent, [^{68}Ga]Ga-DOTA.SA.FAPi, [^{68}Ga]Ga-DOTAGA.(SA.FAPi)2 and ^{68}Ga-labelled colloid remained immobilized at the starting point, whereas free gallium-68 moved with the mobile phase. When the second eluent was used, only the [^{68}Ga]Ga-DOTA.SA.FAPi and [^{68}Ga]Ga-DOTAGA.(SA.FAPi)2 moved with the mobile phase/solvent front.

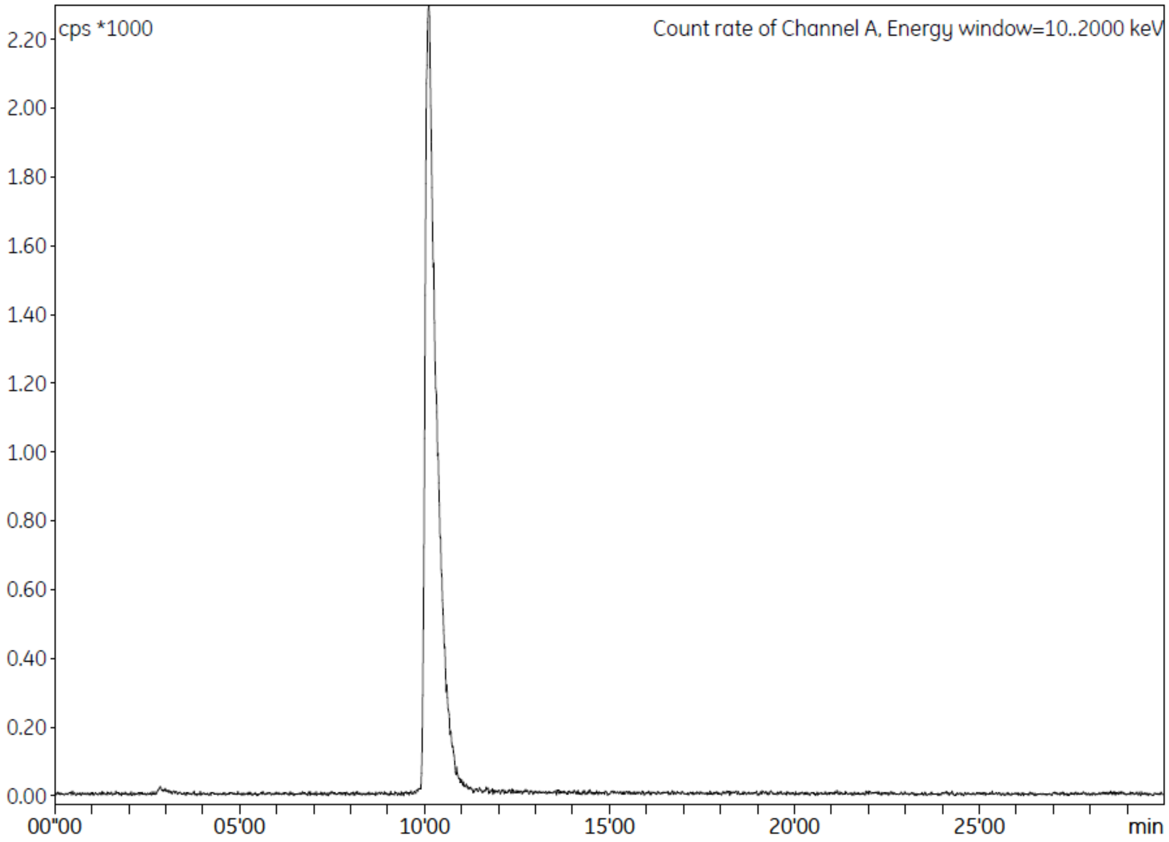

Figure S2. HPLC profile of [^{68}Ga]Ga-DOTA.SA.FAPi.

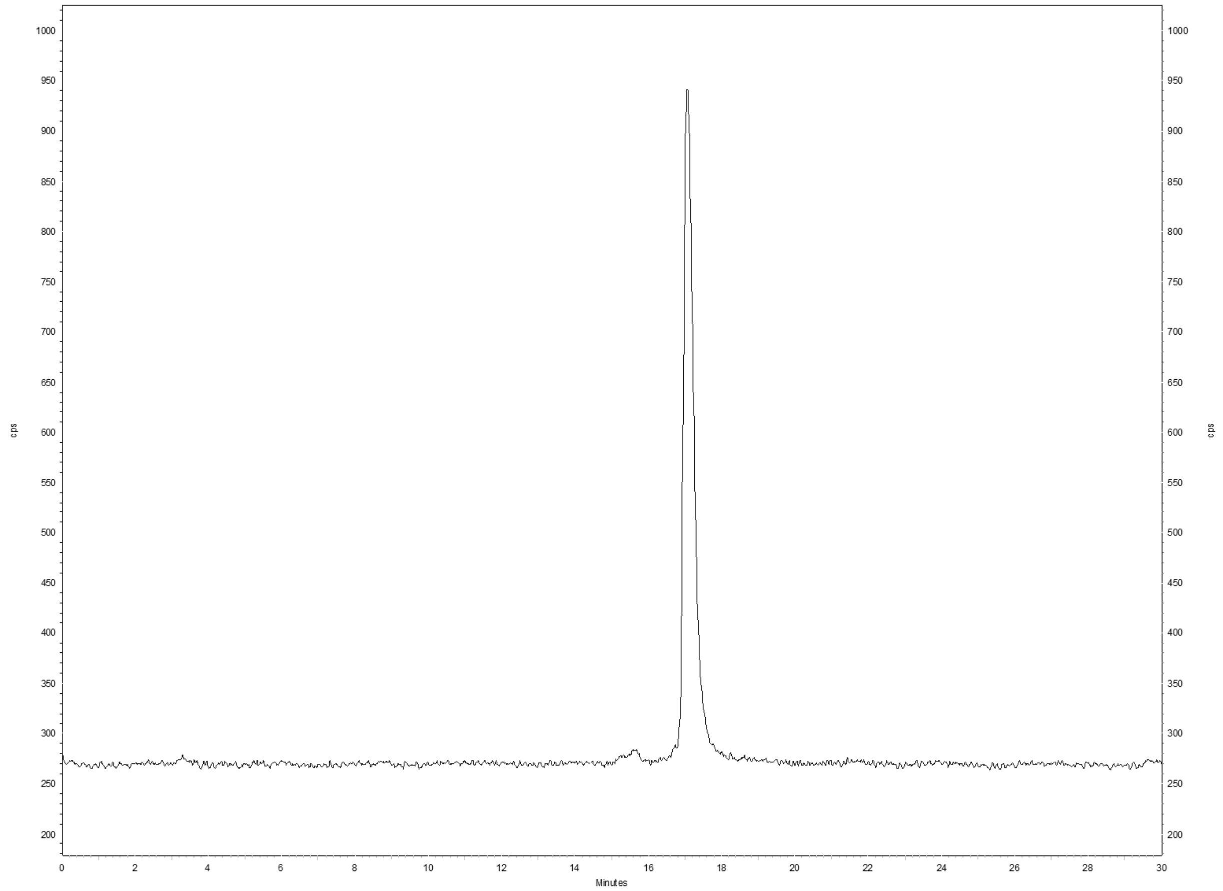

Figure S3. HPLC profile of [^{68}Ga]Ga-DOTAGA.(SA.FAPi)$_2$.

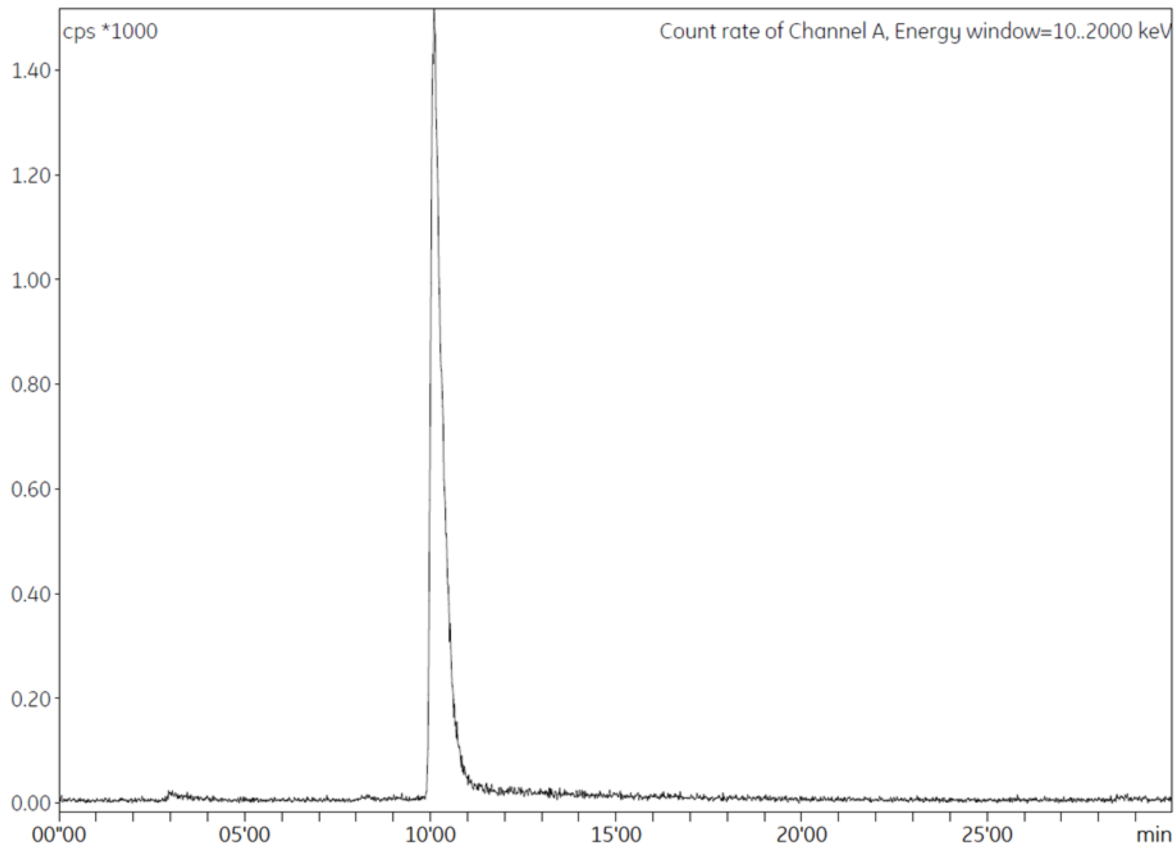

Figure S4. HPLC profile of [^{177}Lu]Lu-DOTA.SA.FAPi.

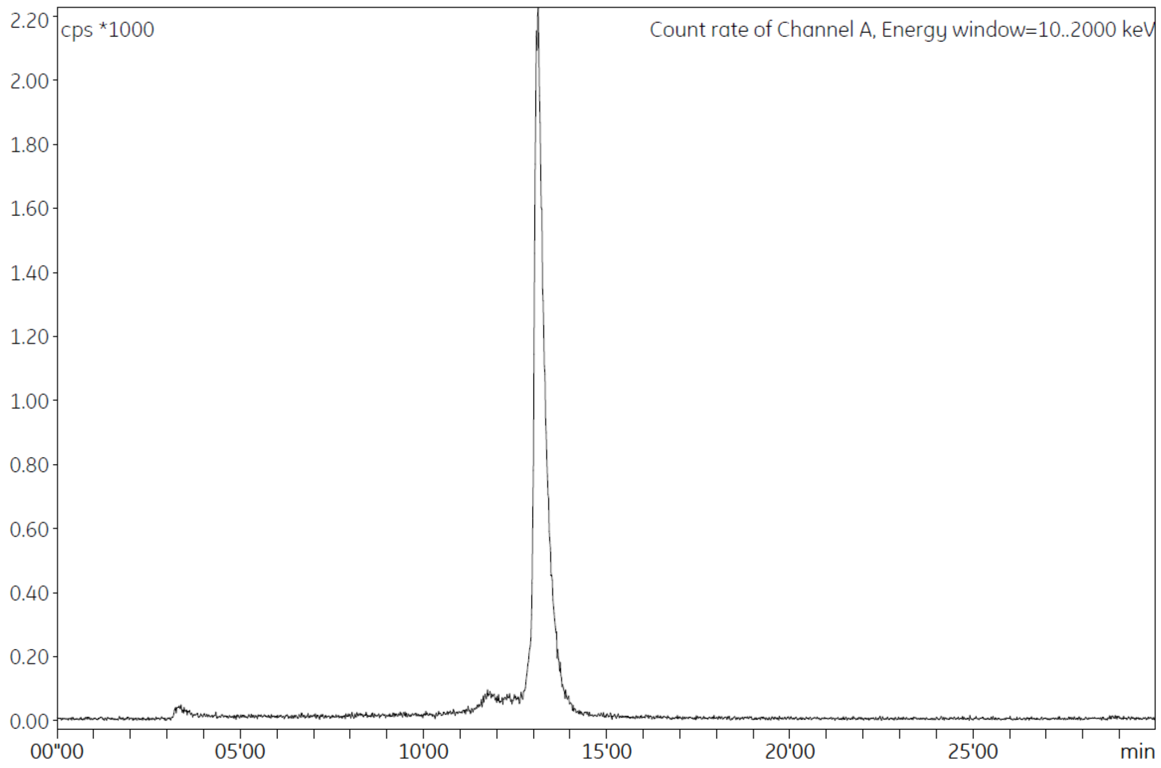

Figure S5. HPLC profile of [^{177}Lu]Lu-DOTAGA.(SA.FAPi)$_2$.

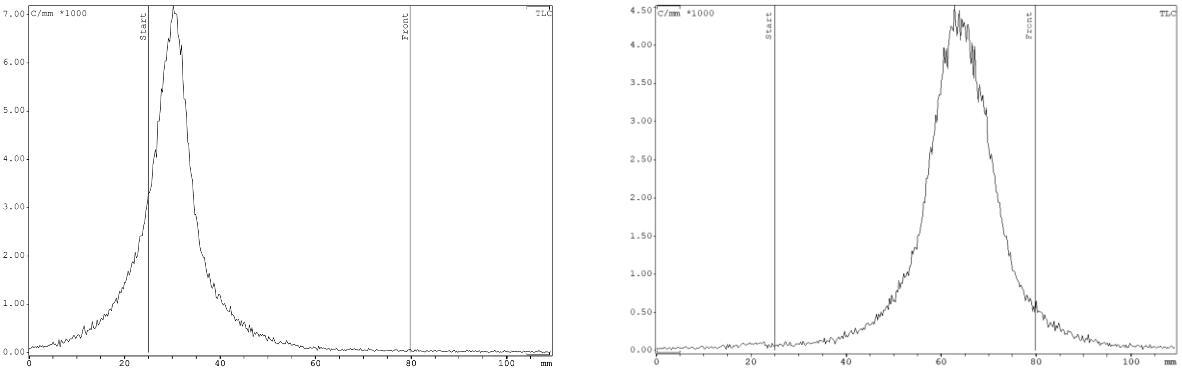

a **b**

Figure S6. Representative TLC profiles in a. sodium citrate and b. ammonium acetate:MeOH of [^{68}Ga]Ga-DOTA.SA.FAPi.

Saturation Binding Studies

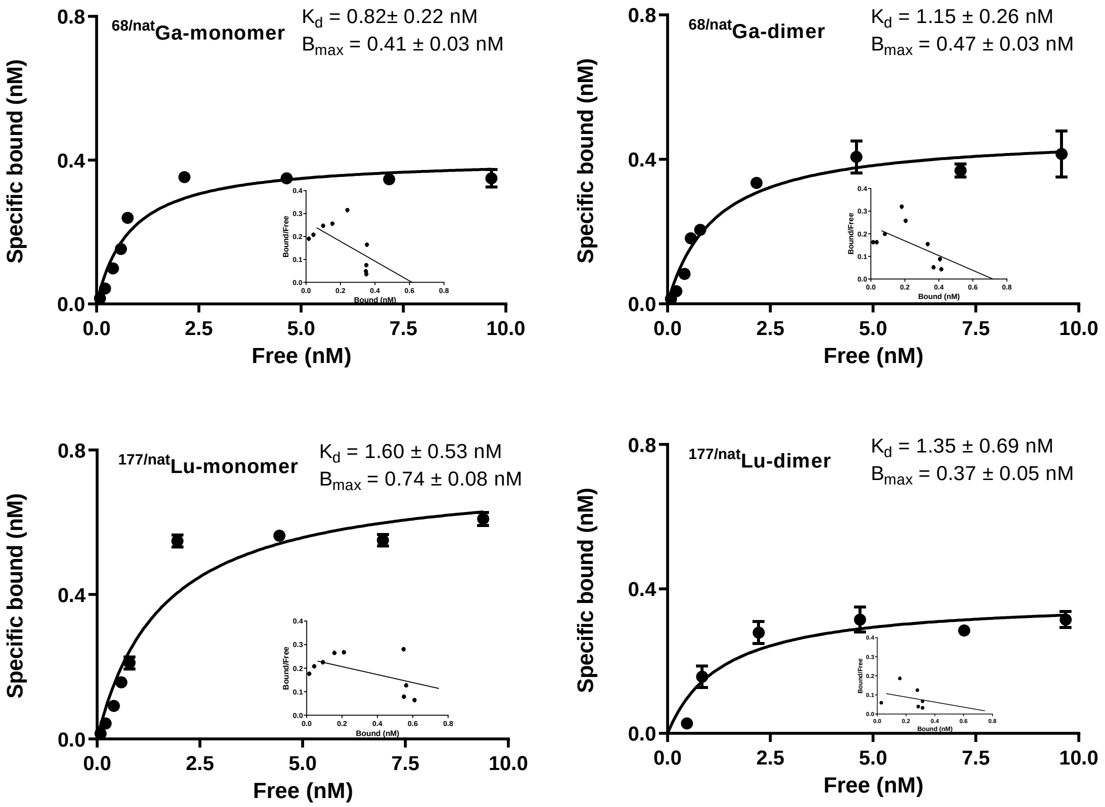

Figure S7. Saturation binding study on CAF cells using increasing concentrations of $^{68/nat}$Ga-DOTA.SA.FAPi ($^{68/nat}$Ga-monomer) and $^{68/nat}$Ga-DOTAGA.(SA.FAPi)$_2$ ($^{68/nat}$Ga-dimer) (0.1 to 10 nM). Dissociation constant (K_d) and maximum number of binding sites (B_{max}) were calculated from nonlinear regression analysis using GraphPad Prism 8.

Internalization Studies

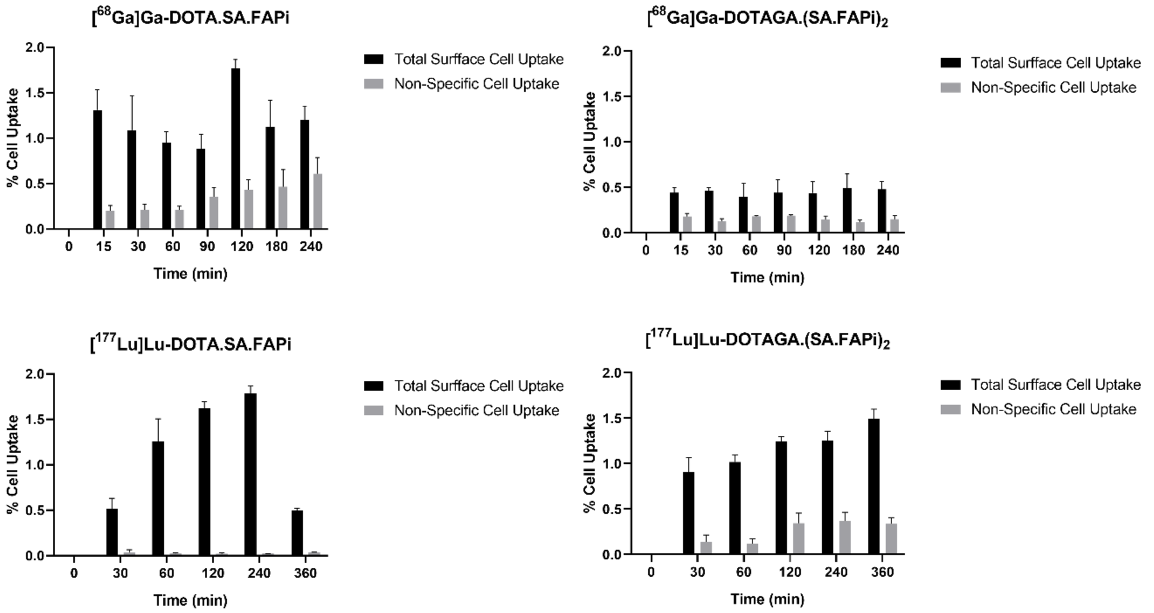

Figure S8. Total surface bound and non-specific cell bound uptake after the incubation of CAFs with [^{68}Ga]Ga-DOTA.SA.FAPi, [^{68}Ga]Ga-DOTAGA.(SA.FAPi)$_2$, [^{177}Lu]Lu-DOTA.SA.FAPi and [^{177}Lu]Lu-DOTAGA.(SA.FAPi)$_2$ within 4 and 6 h at 37 °C. The uptakes are expressed as the percentage of the total applied radioactivity. Nonspecific binding was determined in the presence of 1 μM UAMC1110.

Biodistribution Studies

[^{68}Ga]Ga-DOTA.SA.FAPi and [^{68}Ga]Ga-DOTAGA.(SA.FAPi)$_2$

Table S1. Biodistribution data and tissue radioactivity ratios of [^{68}Ga]Ga-DOTA.SA.FAPi and [^{68}Ga]Ga-DOTAGA.(SA.FAPi)$_2$ on PC3 xenografts.

	[^{68}Ga]Ga-DOTA.SA.FAPi				[^{68}Ga]Ga-DOTAGA.(SA.FAPi)$_2$			
**Organ**	**1 h**	**2 h**	**3 h**	**2 h blocking**	**1 h**	**2 h**	**3 h**	**2 h blocking**
**Blood**	8.5± 0.6	4.8±0.3	4.7±0.4	0.3±0.1	23.3±3.8	11.9±2.8	10.7±0.9	0.7±0.01
**Heart**	4.6±1.6	1.9±0.1	2.0±0.1	0.1±0.05	7.1±0.7	5.0±0.4	4.2±0.3	0.1±0.01
**Liver**	2.4±0.1	2.7±0.2	2.6±0.4	0.2±0.04	8.2±2.1	4.6±0.4	4.6±0.3	1.4±0.2
**Spleen**	2.5±0.1	1.7±0.1	1.8±0.5	0.1± 0.04	4.7±0.5	3.3±0.6	3.0±0.7	0.4±0.2
**Lung**	3.4±0.6	2.2±0.1	2.1±0.3	0.3±0.1	7.1±0.2	4.8±1.1	4.1±0.7	1.5±0.5
**Kidney**	3.6±0.3	2.7±0.2	2.4±0.4	1.2±0.2	8.1±0.6	4.4±0.4	4.2±0.3	3.4±0.07
**Stomach**	2.0±0.4	1.4±0.1	1.6±0.1	0.1±0.07	3.3±0.4	2.8±0.7	2.1±0.3	0.1±0.09
**Intestine**	2.7±0.2	2.6±0.2	2.4±0.4	0.4± 0.1	5.0±0.2	3.5±0.4	3.5±0.03	0.7±0.1
**Pancreas**	8.9±1.0	6.4±0.5	5.1±1.1	0.1±0.06	11.7±1.7	11.4±0.9	9.5±0.2	0.3±0.1
**Muscle**	2.5±0.2	2.6±0.3	2.3±0.2	0.1±0.06	2.4±0.5	1.6±0.3	1.8±0.4	0.3± 0.0
**Bone**	3.4±0.4	2.0±0.2	3.4±2.4	0.2±0.1	4.0±0.3	2.2±1.0	2.6±1.1	0.0 ± 0.0
**PC3-Tumor**	6.1±0.7	5.4±2.4	5.0±0.2	0.5±0.1	6.3±1.0	6.1±0.5	6.3±1.6	0.7±0.1
**Tumor/Blood**	0.7±0.03	1.2±0.6	1.8±0.2		0.3±0.01	0.5±0.1	0.6±0.2	
**Tumor/Liver**	1.8±0.1	2.1±0.1	3.3±0.5		0.9±0.1	1.3±0.4	1.4±0.4	
**Tumor/Muscle**	2.4±0.4	2.1±1.2	3.8±0.4		2.8±0.8	3.9±0.9	3.4±0.3	
**Tumor/Pancreas**	0.7±0.1	0.9±0.4	1.7±0.4		0.6±0.2	0.5±0.1	0.7±0.2	

Data are presented as % IA/g of tissue and are presented as mean ± SD (*n* = 4).

Table S2. Biodistribution data and tissue radioactivity ratios of [^{68}Ga]Ga-DOTA.SA.FAPi and [^{68}Ga]Ga-DOTAGA.(SA.FAPi)$_2$ on U87MG xenografts.

	[^{68}Ga]Ga-DOTA.SA.FAPi				[^{68}Ga]Ga-DOTAGA.(SA.FAPi)$_2$			
**Organ**	**1 h**	**2 h**	**3 h**	**2 h blocking**	**1 h**	**2 h**	**3 h**	**2 h blocking**
**Blood**	13.4 ± 0.2	8.5 ± 1.5	6.2 ± 0.5	0.5 ± 0.1	19.6 ± 4.4	16.8 ± 1.4	16.1 ± 1.4	1.6 ± 0.3
**Heart**	4.9 ± 0.6	3.1 ± 0.9	3.7 ± 0.7	0.7 ± 0.3	9.3 ± 1.9	8.7 ± 2.2	9.2 ± 0.7	1.0 ± 0.2
**Liver**	3.2 ± 0.4	3.1 ± 0.9	3.9 ± 0.3	0.3 ± 0.03	5.6 ± 0.8	6.9 ± 1.3	7.2 ± 0.9	2.0 ± 0.4
**Spleen**	2.3 ± 0.4	1.6 ± 0.5	2.8 ± 1.0	0.7 ± 0.3	4.0 ± 0.2	5.0 ± 1.1	5.9 ± 0.2	0.9 ± 0.2
**Lung**	3.2 ± 1.7	2.2 ± 1.0	3.1 ± 1.0	1.1 ± 0.2	5.3 ± 3.2	6.6 ± 0.7	7.2 ± 1.1	2.9 ± 0.2
**Kidney**	3.5 ± 0.7	2.7 ± 0.6	3.4 ± 0.3	2.5 ± 0.8	6.9 ± 1.1	6.7 ± 0.9	7.9 ± 0.4	5.5 ± 1.0
**Stomach**	2.1 ± 0.6	2.0 ± 0.5	2.2 ± 0.5	0.7 ± 0.4	3.4 ± 1.1	3.1 ± 0.8	4.8 ± 0.6	1.0 ± 0.2
**Intestine**	2.9 ± 0.5	3.2 ± 0.7	3.5 ± 0.7	1.3 ± 0.1	4.5 ± 0.8	4.4 ± 0.6	4.9 ± 0.5	2.3 ± 1.1
**Pancreas**	9.7 ± 2.7	10.5 ± 2.8	8.7 ± 1.3	0.6 ± 0.4	12.6 ± 5.7	11.3 ± 2.3	11.6 ± 1.4	1.5 ± 0.4
**Muscle**	2.6 ± 1.4	2.8 ± 1.1	3.1 ± 0.9	1.4 ± 0.8	4.3 ± 1.0	3.7 ± 0.1	4.5 ± 0.6	0.7 ± 0.2
**Bone**	2.1 ± 0.2	1.3 ± 0.2	3.0 ± 1.6	1.7 ± 0.6	5.0 ± 1.1	5.6 ± 1.0	4.6 ± 2.3	2.1 ± 1.3
**U87MG-Tumor**	6.5 ± 0.7	5.2 ± 1.4	4.9 ± 1.0	1.1 ± 0.8	7.1 ± 0.6	9.0 ± 1.4	9.4 ± 0.6	2.9 ± 0.04
**Tumor/Blood**	0.5 ± 0.03	0.7 ± 0.3	0.8 ± 0.3		0.4 ± 0.1	0.5 ± 0.06	0.6 ± 0.1	
**Tumor/Liver**	2.0 ± 0.3	1.7 ± 0.5	1.3 ± 0.5		1.6 ± 0.7	1.4 ± 0.4	1.3 ± 0.2	
**Tumor/Muscle**	2.3 ± 0.7	1.9 ± 1.5	1.7 ± 1.2		1.7 ± 0.3	2.4 ± 0.4	2.1 ± 0.3	
**Tumor/Pancreas**	0.7 ± 0.2	0.5 ± 0.2	0.6 ± 0.3		0.6 ± 0.3	0.8 ± 0.1	0.8 ± 0.1	

Data are presented as % IA/g of tissue and are presented as mean ± SD (*n* = 4).

Table S3. Biodistribution Data of [^{68}Ga]Ga-DOTA.SA.FAPi in Healthy Male and Female Mice at 1 h p.i.

	[^{68}Ga]Ga-DOTA.SA.FAPi	
**Organ**	**Male**	**Female**
**Blood**	8.5 ± 0.1	13.5 ± 4.1
**Heart**	3.1 ± 0.04	5.1 ± 0.9
**Liver**	2.3 ± 0.1	3.3 ± 0.4
**Spleen**	2.3 ± 0.1	2.7 ± 0.7
**Lung**	2.7 ± 0.6	4.3 ± 1.6
**Kidney**	3.5 ± 0.4	5.0 ± 0.7
**Stomach**	2.0 ± 0.3	1.7 ± 0.04
**Intestine**	3.1 ± 0.2	3.0 ± 0.3
**Pancreas**	8.6 ± 0.1	7.6 ± 2.6
**Muscle**	2.0 ± 0.3	6.3 ± 3.4
**Bone**	1.5 ± 0.9	5.0 ± 0.2

Data are presented as % IA/g of tissue and are presented as mean ± SD (n = 4).

[^{177}Lu]Lu-DOTA.SA.FAPi and [^{177}Lu]Lu-DOTAGA.(SA.FAPi)$_2$

Table S4. Biodistribution data and tissue radioactivity ratios of [^{177}Lu]Lu-DOTA.SA.FAPi and [^{177}Lu]Lu-DOTAGA.(SA.FAPi)$_2$ on PC3 xenografts.

	**[^{177}Lu]Lu-DOTA.SA.FAPi**				**[^{177}Lu]Lu-DOTAGA.(SA.FAPi)$_2$**				
**Organ**	**1 h**	**4 h**	**24 h**	**48 h**	**4 h**	**24 h**	**48 h**	**72 h**	**96 h**
**Blood**	9.1 ± 1.8	3.1 ± 0.2	0.2 ± 0.09	0.01 ± 0.0	11.8 ± 0.8	3.8 ± 0.6	1.0 ± 0.2	0.3 ± 0.1	0.1 ± 0.01
**Heart**	3.1 ± 0.7	1.5 ± 0.3	0.1 ± 0.04	0.03 ± 0.02	4.4 ± 0.1	2.7 ± 0.1	1.4 ± 0.05	1.2 ± 0.4	0.7 ± 0.1
**Liver**	3.8 ± 0.2	3.0 ± 0.1	1.9 ± 0.3	1.1 ± 0.1	7.4 ± 2.3	10.4 ± 1.0	8.1 ± 0.9	8.1 ± 1.7	5.6 ± 0.2
**Spleen**	3.0 ± 0.1	1.7 ± 0.1	0.2 ± 0.03	0.1 ± 0.06	4.7 ± 0.5	4.8 ± 0.6	3.3 ± 0.5	3.1 ± 0.8	2.4 ± 0.4
**Lung**	4.3 ± 0.6	1.7 ± 0.07	0.2 ± 0.03	0.05 ± 0.04	4.7 ± 1.3	2.3 ± 0.6	1.0 ± 0.2	0.7 ± 0.2	0.4 ± 0.04
**Kidney**	4.3 ± 0.5	2.6 ± 0.03	0.6 ± 0.06	0.12 ± 0.07	6.6 ± 0.4	5.7 ± 0.3	3.7 ± 0.1	2.9 ± 1.3	1.4 ± 0.1
**Stomach**	2.5 ± 0.3	1.7 ± 0.5	0.1 ± 0.02	0.05 ± 0.02	2.3 ± 0.3	1.5 ± 0.1	0.8 ± 0.2	0.5 ± 0.1	0.4 ± 0.0
**Intestine**	3.2 ± 0.4	1.8 ± 0.3	0.2 ± 0.02	0.04 ± 0.0	3.6 ± 1.0	1.9 ± 0.4	0.9 ± 0.1	0.7 ± 0.3	0.3 ± 0.01
**Pancreas**	12 ± 1.1	5.3 ± 0.4	0.3 ± 0.1	0.06 ± 0.01	8.4 ± 0.8	4.7 ± 0.4	2.5 ± 0.3	1.8 ± 0.7	0.8 ± 0.1
**Muscle**	3.7 ± 1.0	2.3 ± 0.1	0.3 ± 0.04	0.05 ± 0.07	2.1 ± 0.2	1.3 ± 0.1	0.6 ± 0.1	0.6 ± 0.3	0.2 ± 0.1
**Bone**	4.0 ± 0.5	4.2 ± 0.6	0.7 ± 0.2	0.1 ± 0.01	6.7 ± 1.1	5.7 ± 0.6	3.2 ± 0.6	3.3 ± 0.7	2.2 ± 0.1
**PC3-Tumor**	9.6 ± 0.9	8.9 ± 0.2	1.8 ± 0.1	1.0 ± 0.1	8.6 ± 0.7	5.6 ± 0.6	3.9 ± 0.5	2.6 ± 0.9	1.8 ± 0.1
**Tumor/Blood**	1.1 ± 0.16	2.9 ± 0.2			0.7 ± 0.1	1.5 ± 0.2	3.8 ± 0.6	8.2 ± 0.7	
**Tumor/Liver**	2.2 ± 0.2	3.0 ± 0.1			1.2 ± 0.4	0.5 ± 0.1	0.5 ± 0.1	0.3 ± 0.04	
**Tumor/Muscle**	2.8 ± 1.0	3.9 ± 0.1			4.1 ± 0.5	4.3 ± 0.3	6.6 ± 0.8	4.8 ± 0.7	
**Tumor/Pancreas**	0.8 ± 0.1	1.7 ± 0.2			1.0 ± 0.1	1.2 ± 0.1	1.6 ± 0.1	1.5 ± 0.2	

Data are presented as % IA/g of tissue and are presented as mean ± SD (n = 4).

Small-Animal PET/SPECT/CT Studies

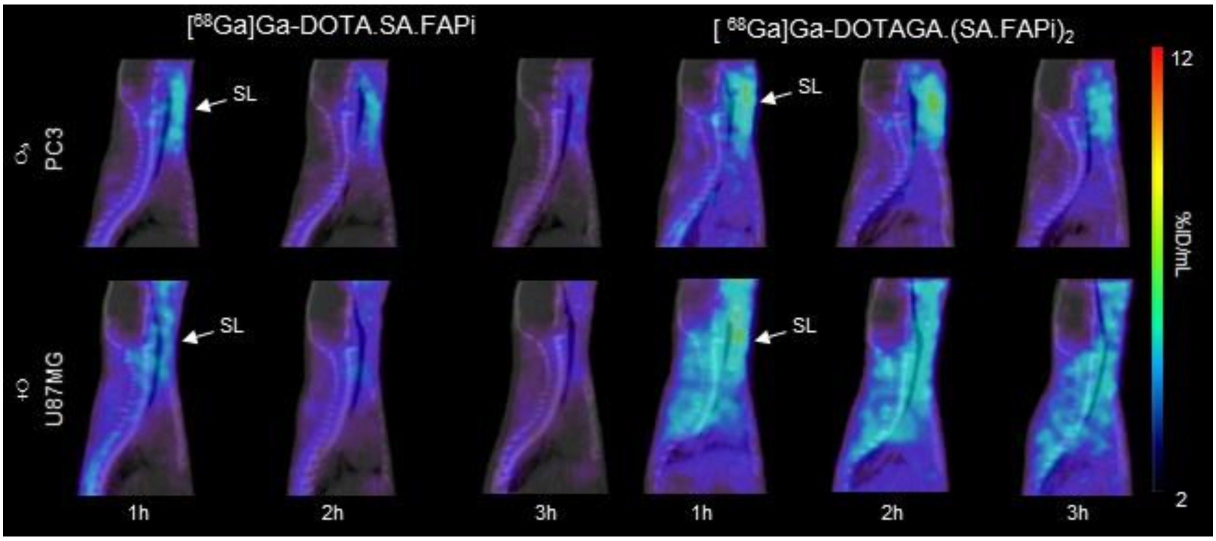

Figure S9. Representative slices (thickness 0.25 mm) of PET/CT images of PC3 (male) and U87MG (female) xenografts upon injection of [^{68}Ga]Ga-DOTA.SA.FAPi and [^{68}Ga]Ga-DOTAGA.(SA.FAPi)$_2$ at 1, 2 and 3 h after injection (SL: salivary glands).
